# *Streptococcus pneumoniae’s* Virulence and Host Immunity: Aging, Diagnostics, and Prevention

**DOI:** 10.3389/fimmu.2018.01366

**Published:** 2018-06-22

**Authors:** Lavida R. K. Brooks, George I. Mias

**Affiliations:** ^1^Microbiology and Molecular Genetics, Michigan State University, East Lansing, MI, United States; ^2^Institute for Quantitative Health Science & Engineering, Michigan State University, East Lansing, MI, United States; ^3^Biochemistry and Molecular Biology, Michigan State University, East Lansing, MI, United States

**Keywords:** pneumococcal, pathogenesis, respiratory, immunology, virulence

## Abstract

*Streptococcus pneumoniae* is an infectious pathogen responsible for millions of deaths worldwide. Diseases caused by this bacterium are classified as pneumococcal diseases. This pathogen colonizes the nasopharynx of its host asymptomatically, but overtime can migrate to sterile tissues and organs and cause infections. Pneumonia is currently the most common pneumococcal disease. Pneumococcal pneumonia is a global health concern and vastly affects children under the age of five as well as the elderly and individuals with pre-existing health conditions. *S. pneumoniae* has a large selection of virulence factors that promote adherence, invasion of host tissues, and allows it to escape host immune defenses. A clear understanding of *S. pneumoniae’s* virulence factors, host immune responses, and examining the current techniques available for diagnosis, treatment, and disease prevention will allow for better regulation of the pathogen and its diseases. In terms of disease prevention, other considerations must include the effects of age on responses to vaccines and vaccine efficacy. Ongoing work aims to improve on current vaccination paradigms by including the use of serotype-independent vaccines, such as protein and whole cell vaccines. Extending our knowledge of the biology of, and associated host immune response to *S. pneumoniae* is paramount for our improvement of pneumococcal disease diagnosis, treatment, and improvement of patient outlook.

## Introduction

**Figure 1:**
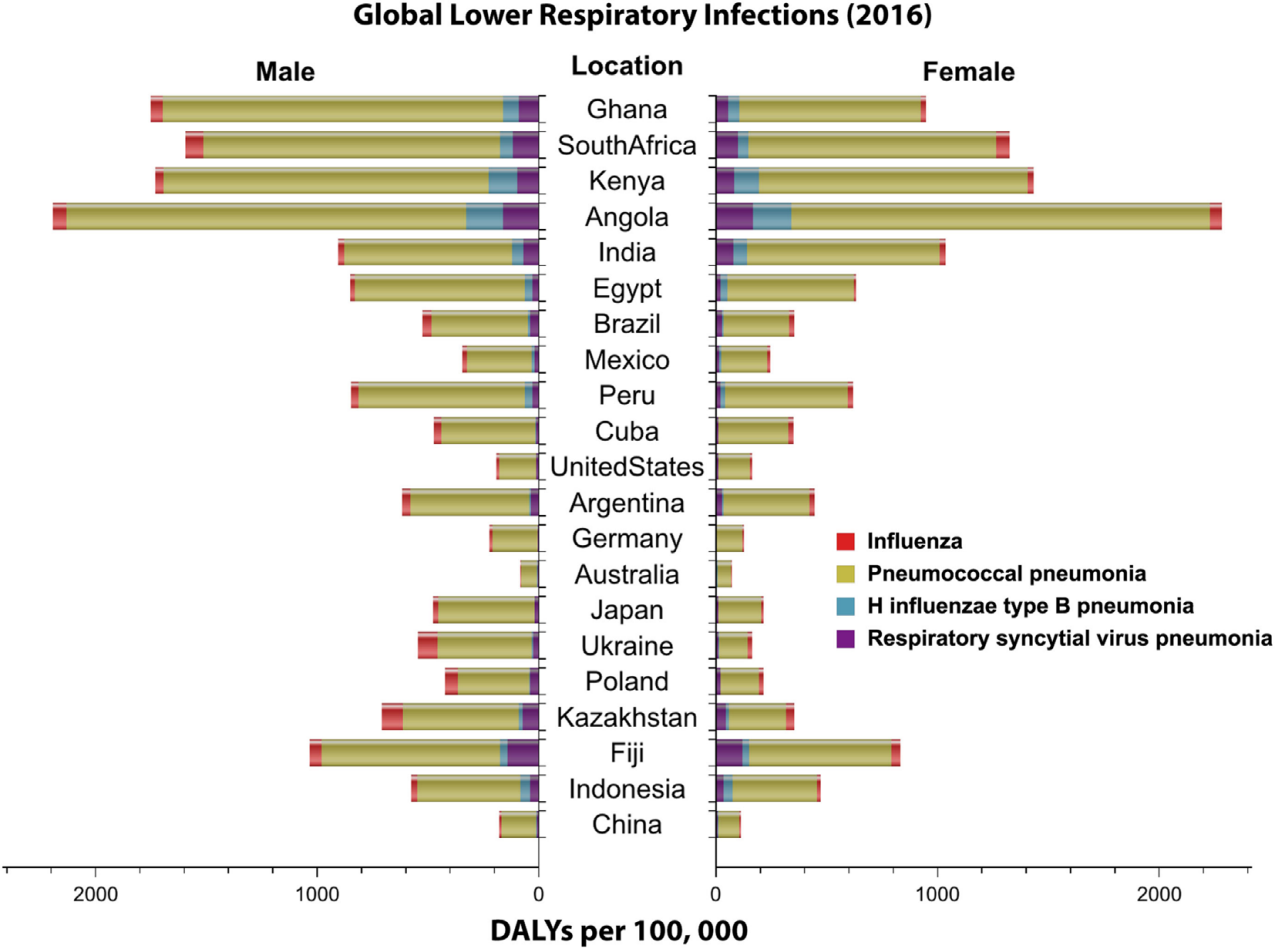
Global distribution of lower respiratory infections by sex. Highlighted in this figure is the distribution of the disability adjusted life year (DALY) per 100,000 (2016) for four major lower respiratory infections worldwide by sex. Data obtained from Institute for Health Metrics and Evaluation (7).

Infectious diseases present a significant global burden affecting society (1, 2). Most of these diseases are due to exposure to or the invasion of host cells and organs by microorganisms (1–3). These pathogens disrupt the normal function of the human body by hindering immune responses and producing harmful toxins. Infectious diseases can easily spread from person-to-person *via* contact with body fluids, indirect contact or through animal vectors such as mosquitoes and ticks (4). Common widespread diseases of the respiratory system occur when microorganisms invade the respiratory tract. Infectious respiratory diseases are globally seen as a major health concern because they can rapidly become severe and lead to death. Respiratory diseases are categorized into upper and lower respiratory tract infections (LRIs). LRIs are more severe because pathogens infect sterile parts of the respiratory system such as the lungs, trachea, and bronchi (5). In 2013, an estimated 2.6 million deaths worldwide were attributed to LRIs, while by 2015, this increased to 2.74 million (6). Higher burden of LRIs is associated with low sociodemographic status, poor access to healthcare and nutrition (Figure 1) (6, 7).

Immune system function is important in a host’s defense to pathogens. A host with a healthy and well-developed immune system is able to clear pathogens before they can become infectious and cause diseases (8–11). The ability to clear pathogens before they can become infectious depends on the quality of the immune system and its effectiveness, which is linked strongly to age (8, 12). The immune system continues to develop from infancy to adulthood, while later in life a fully developed immune system begins to deteriorate with aging. Infants and the elderly are at higher risks for contracting infectious diseases due to their weakened immune system and the inability to clear the pathogens before they become pathogenic (8–11, 13–17).

**Figure 2:**
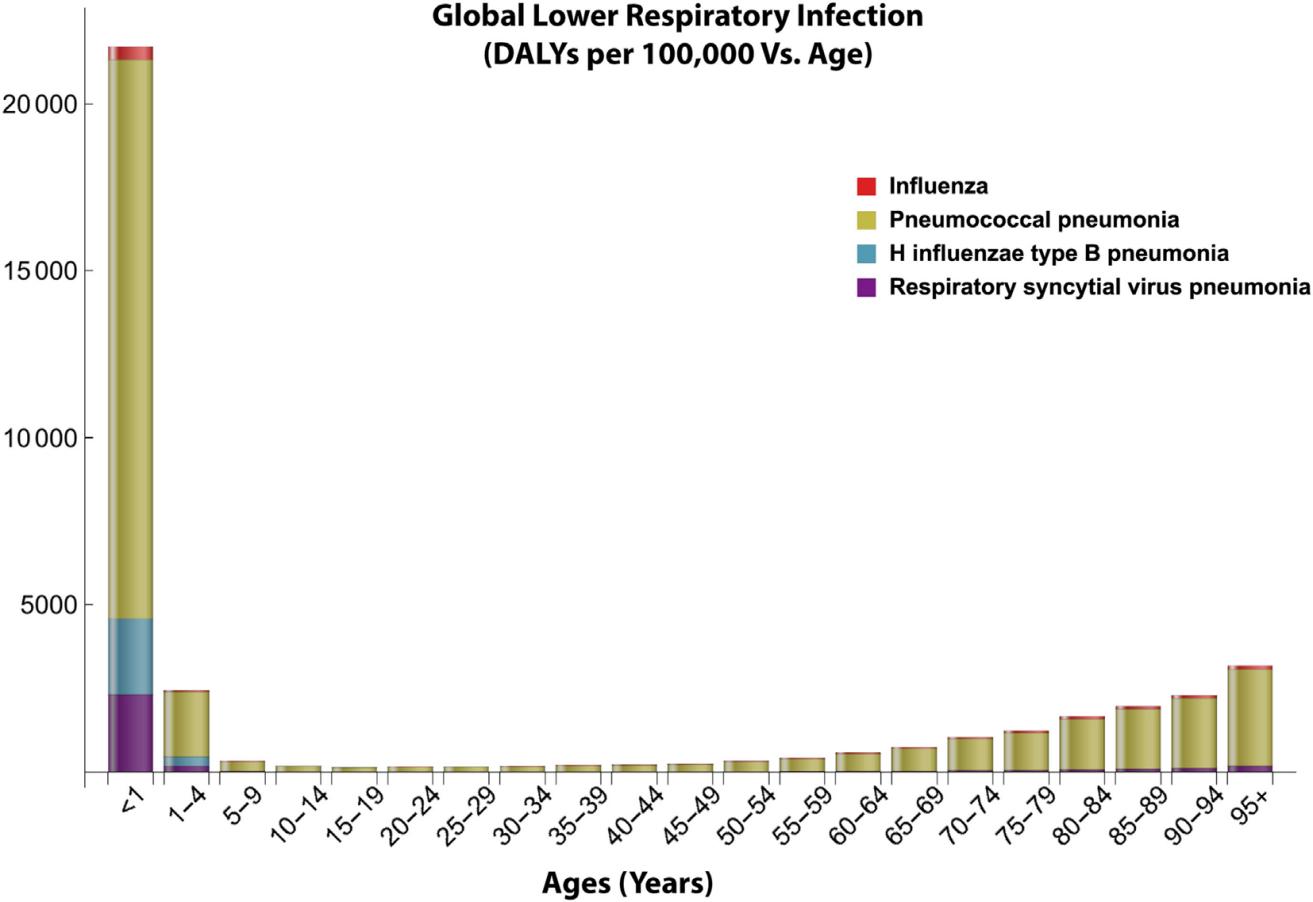
Global distribution of lower respiratory infections with age. This figure shows the age-dependent disease burden to lower respiratory infections especially pneumococcal pneumonia based on the disability adjusted life year (DALY) data from 2016. Data obtained from Institute for Health Metrics and Evaluation (7).

*Streptococcus pneumoniae* is a bacterium that has been widely linked to causing respiratory infections in individuals with a weakened immune system (9, 12, 16). *S. pneumoniae* is spread through airborne droplets, and it is estimated to cause about four million illnesses within the United States (US) and about 450,000 hospitalizations per year (18, 19). Studies indicate that 10% of patients with invasive pneumococcal diseases die of their illnesses (20, 21). *S. pneumoniae* invades its host by colonizing the nasopharynx asymptomatically as it has been found to be part of the commensal microbiota of the upper respiratory tract (9, 22). After colonization, if the bacterium is not cleared by the immune system, the bacterium is spread *via* horizontal dissemination into the lower airways and other organs and tissues, and becomes pathogenic (22). A strong immune system and the balance between resident flora and invaders can help to clear *S. pneumoniae* before it becomes pathogenic. With poor defense mechanisms, the host becomes subject to frequent and long-lasting colonization of *S. pneumoniae*, which can later lead to diseases (23, 24). The bacterium has several properties which allow it to go unnoticed by the host immune system, and defend against the resident flora within the nasopharynx that would try to clear it (17, 25, 26). Thus, decreasing the burden of this bacterium and preventing further infections is very important to the healthcare field (26, 27). Furthermore, *S. pneumoniae* is an opportunistic pathogen that takes advantage of hosts with underdeveloped, weakened, and or deteriorating immune systems. Because of this, *S. pneumoniae* has greater incidence rates in children under the age of two, the immunocompromised, and the elderly (28). Figure 2 depicts that disease burden for major LRIs are highest in young children and the elderly (7, 20, 29–31). Understanding how the immune system changes with age is important in providing appropriate treatments to hinder colonization of weaker hosts.

In this review, we provide a concise introduction to the expanding literature on *S. pneumoniae*, and focus on exploring the characteristics of *S. pneumoniae*, its pathogenesis, its virulence factors, and pathology. We will also delve into the general host immune response to *S. pneumoniae*, with a focus on pneumonia, and connect the severity of this disease to varying host immune responses with age. In addition, we will explore the medications available to prevent or treat pneumococcal diseases such as pneumonia, disease prognosis, and finally discuss what the future holds for pneumococcal diseases.

## Pneumococcal Disease, Epidemiology, and Transmission

**Figure 3:**
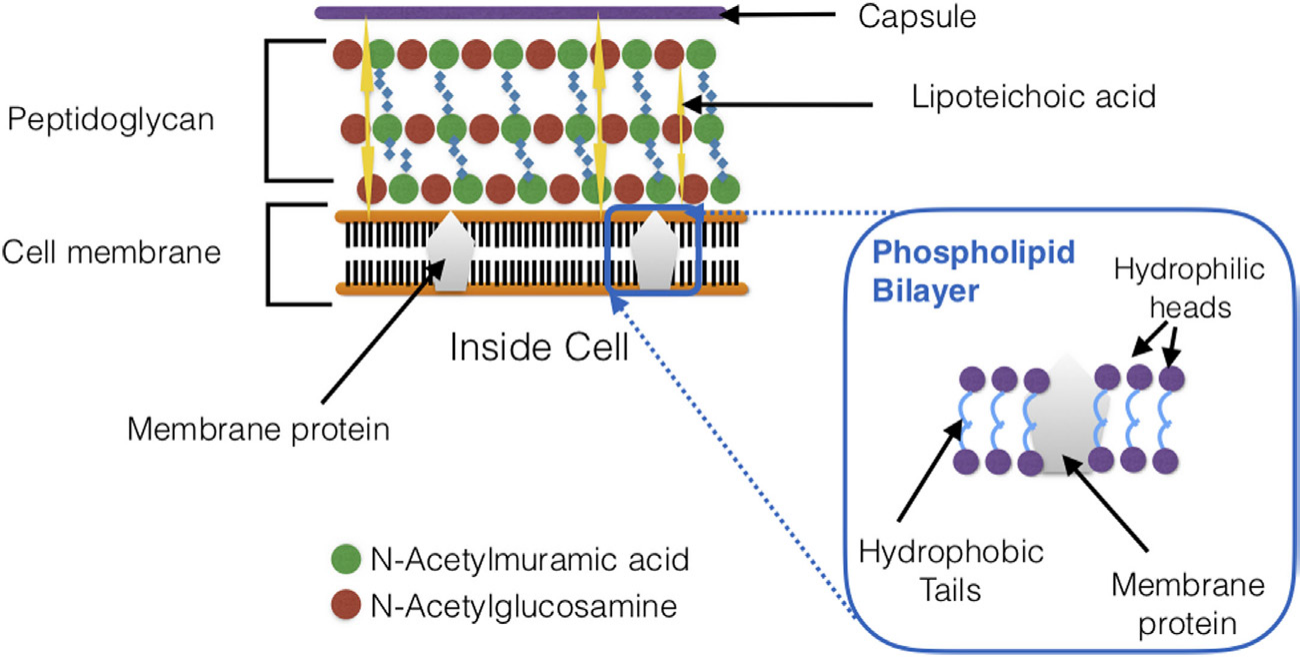
Schematic cross section of *Streptococcus pneumoniae* cell wall. The bacterial cell wall composes of teichoic acids, a thick peptidoglycan layer, and a phospholipid bilayer.

*Streptococcus pneumoniae*, a Gram-positive bacterium (Figure 3), also known as pneumococcus, can survive in both aerobic and anaerobic conditions (32). It is a facultative anaerobe that is often found as diplococci (32). Pasteur and Sternberg first isolated *S. pneumoniae* from saliva in 1881 (33–35). Currently, there are varying reports on the number of identified serotypes of *S. pneumoniae* (24, 34, 36, 37). However, there are at least 97 serotypes of *S. pneumoniae* that have been identified and characterized to date (34, 38). All of these serotypes are independently recognized by the host (9, 24, 39–41).

**Figure 4:**
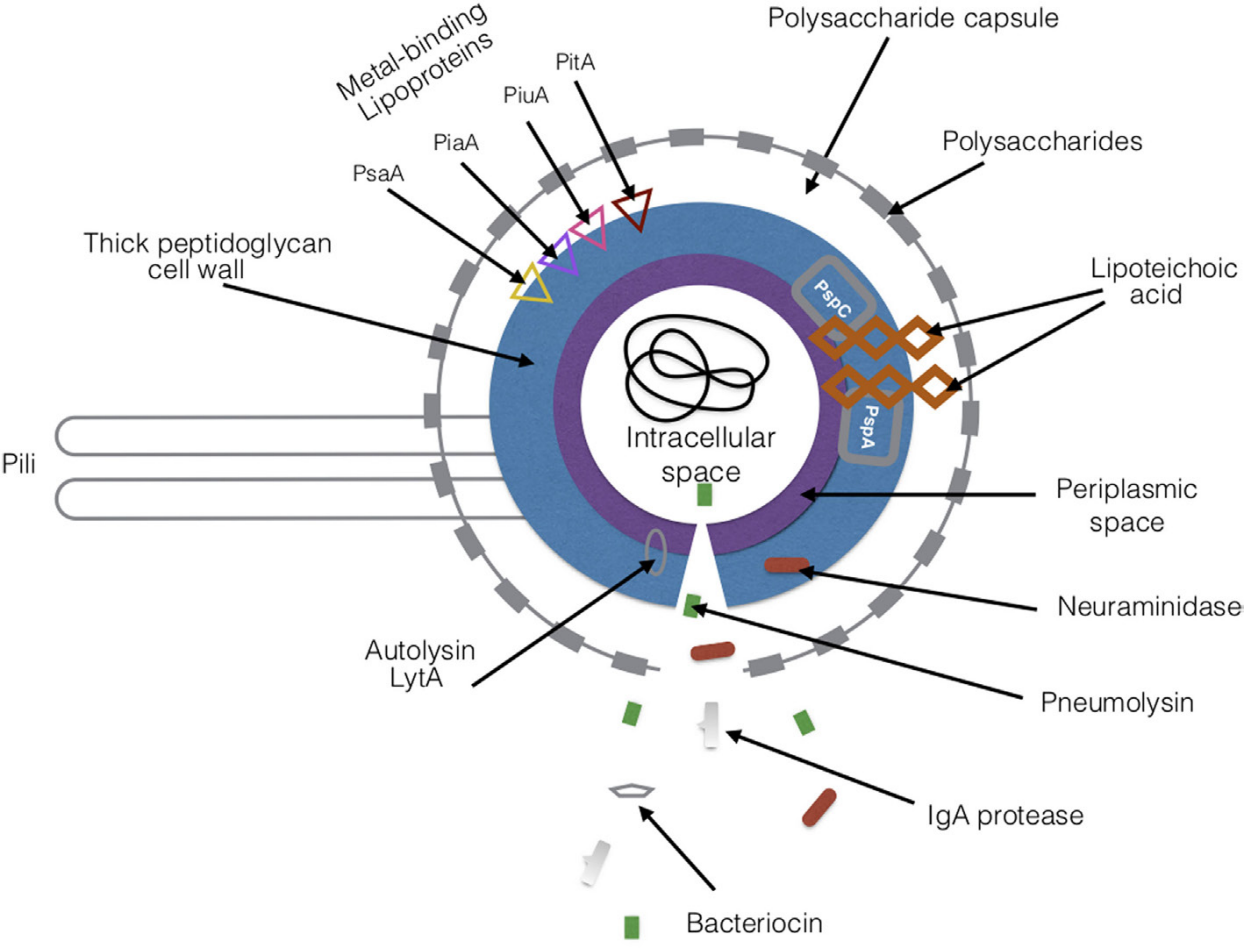
Virulence factors of *Streptococcus pneumoniae*. There are a variety of proteins and toxins that are expressed by *S. pneumoniae* that drive its pathogenesis. The major virulence factors are highlighted in the figure. Abbreviations: PsaA, pneumococcal surface adhesin A; PspA, pneumococcal surface protein A; PspC, pneumococcal surface protein C; PiaA, pneumococcal iron acquisition A; PiuA, pneumococcal iron uptake A; PitA, pneumococcal iron transporter.

Pneumococcal diseases occur worldwide (24, 26, 42) and are more prevalent in young children, the elderly, and immunocompromised individuals (Table 1) (9, 22, 32, 41, 43, 44). *S. pneumoniae* causes many pneumococcal diseases such as meningitis, bacteremia, pneumonia, acute otitis media, and sinusitis (24). *S. pneumoniae* causes about 40,000 fatal pneumococcal infections per year within the US (23, 32, 45, 46). *S. pneumoniae* colonizes the upper respiratory tract—specifically the nasopharynx (9, 47), and is able to asymptomatically reside in the upper respiratory tract—this is known as carriage (9). Carriage is more prevalent in children (20–50%) compared to adults (5–20%) (47–49). Carriage can lead to further transmission of *S. pneumoniae* within the community or can advance to pneumococcal diseases (9). Biofilms form in the nasopharynx during colonization (50). *S. pneumoniae* has many virulence factors (Table 2; Figure 4) that allow for adherence to host cells, reduce the host’s immune system’s ability to clear the bacterium, and promote invasion of epithelial cells (17). If the host is unable to clear *S. pneumoniae* immediately after colonization of the upper respiratory tract, the bacterium multiplies, disrupts the regular non-pathogenic flora of the respiratory system (22, 51), and is able to migrate to the tissues and organs and cause infections. The migration of *S. pneumoniae* to sterile tissues and organs is the main cause of all pneumococcal diseases. For example, when meninges, the protective membranes surrounding the spinal cord and brain, become inflamed due to *S. pneumoniae* infection, this is known as bacterial meningitis (24, 52). Bacterial meningitis is predominantly seen in young children and is mostly caused by *S. pneumoniae* (53). *S. pneumoniae* causes more than 50% of bacterial meningitis within the US (24, 53). Bacteremia refers to infection of the blood by pneumococcus (24) which causes about 12,000 cases per year and usually accompanies other pneumococcal infections (24). *S. pneumoniae* can also colonize the middle ear of infants and young children causing acute otitis media (24). The Centers for Disease Control (CDC) estimates that approximately 60% of young children would have at least one ear infection (24). Sinusitis occurs when *S. pneumoniae* infects fluid trapped in the sinuses (24).

**Table 1:** Occurrence of pneumococcal diseases from 1995 to 2015 as reported^a^ by the Centers for Disease Control.

Year	1997		2007		2012		2014		2015	
Age	Cases rate	Deaths rate	Cases rate	Deaths rate	Cases rate	Deaths rate	Cases rate	Deaths rate	Cases rate	Deaths rate
<1	142.9	4.02	40.51	0.9	15.7	0.24	15.9	0.48	18.4	0.24
1	178.7	0.9	32.39	0.23	13.6	0.24	10.3	0	12.9	0.24
2–4	31	0.15	13.03	0.08	5.9	0	6.3	0.08	5.1	0.16
5–17	4.8	0.14	2.91	0.14	1.9	0.14	1.4	0.05	1.3	0
18–34	9.3	0.52	4.19	0.22	2.8	0.1	2.7	0.18	2.5	0.08
35–49	18.9	1.65	11.89	0.98	7.5	0.6	6.6	0.7	6.7	0.5
50–64	23.5	2.72	20.59	2.33	15.9	1.53	15.1	1.64	15	1.53
65–74	61.7	11.02	39.26	6.37	29.6	4.24	19.1	2.41	18.2	2.3
75–84							28.2	3.46	29	4.5
≥85							42.6	8.01	45.3	11.56

*^a^Rates are per 100,000 population for Active Bacterial Core surveillance areas*.

**Table 2:** Selected virulence factors of *S. pneumoniae*, their location, and function.

Virulence factor	Location on S. pneumoniae	Function	Reference
Polysaccharide capsule	Layer of polysaccharides on cell wall	Allows the bacteria to escape the nasal mucus Inhibits phagocytosis by innate immune cells Escapes neutrophil net traps Inhibits complement and recognition by immunoglobulins Allows adherence and colonization of the nasopharynx	(17, 22, 26, 27, 38, 51, 54–58)

Pneumolysin	Cytoplasmic toxin	Binds to membranes with cholesterol Forms pores which cause cell lysis Induces inflammation Drives host-to-host transmission Can activate complement and modulate chemokine and cytokine production	(22, 26, 27, 51, 56, 59–66)

Autolysin (lytic amidase)	Intracellular enzyme produced by Gram-positive bacteria	Cell lysis Break down peptidoglycan Exposes hosts cell to pneumolysin and teichoic acid Aids with bacterial colonization	(17, 27, 51, 67–70)

Pneumococcal surface protein A	Bound to the cell wall *via* phosphorylcholine (PCho) moiety	Protects against complement system of the host Aids in colonization by adhering to epithelial cell membranes Decreases the deposition of the complement	(17, 22, 38, 51, 60, 71–75)

Pneumococcal surface protein C also known as choline-binding protein A (CbpA)	Bound to the cell wall *via* PCho moiety	Protects against the complement system of the host Binds to receptors such as the human polymeric immunoglobulin A (IgA) during colonization and invasion the nasopharynx Cell adhesion and colonization of nasopharynx	(17, 22, 27, 38, 51, 58, 60, 71–77)

Pneumococcal surface adhesin A (PsaA)	Surface of the cell wall	Transports magnesium and zinc into the cytoplasm of the bacteria Aids in invasion of epithelial cells during nasopharynx colonization	(17, 22, 38, 51, 60, 71–75)

Other choline-binding proteins: LytB, LytC, CbpC, CbpG	Bound to the cell wall *via* PCho moiety	Promote bacterial colonization of the nasopharynx Modify proteins on cell surfaces and allows for binding to host cell receptors Important for host cell recognition	(22, 27, 51, 58, 72, 76, 77)

Non-classical surface proteins	Surface of the cell wall	Act as adhesins Promote immune system evasion by inhibiting complement Controls inflammation and affects cytokine production	(78–80)

Pili	Cell surface	Promotes adherence and colonization of the epithelial cells within the nasopharynx INHIBITS phagocytosis by immune cells	(22, 27, 51, 81, 82)

Bacteriocin	Produced and secreted by the organism	Inhibits the growth of competing bacterial cells	(22, 27, 51)

Neuraminidase	Cell wall bound	Degrades mucus Promotes growth and survival Aids with cell adherence	(22, 27, 51)

Biofilm		Helps to reduce bacterial recognition by the host immune system Reduces the impact of antimicrobial agents on bacteria	(22, 27, 51)

IgA protease	Secreted by the bacteria into the extracellular environment	Breaks down IgA	(22, 27, 51, 83–85)

Lipoteichoic acid	Membrane bound	Causes inflammation	(22, 27, 51)

*Streptococcus pneumoniae*, which initially inhabits the mucosal surfaces of the nasopharynx in its hosts (17), can migrate to the lungs, where it causes pneumococcal pneumonia (17). This is an infection of the lungs that leads to inflammation of the air sacs causing them to fill with fluid, and making it difficult to breathe. Individuals who have pneumonia usually suffer with high heart rates, shortness of breath, frequent coughing, and high fevers (86). Thus, despite *S. pneumoniae’s* asymptomatic colonization of the nasopharynx, having a poor immune response and lack of clearance, may develop into pneumococcal pneumonia, which can be a serious health risk for those with reduced host defenses.

**Figure 5:**
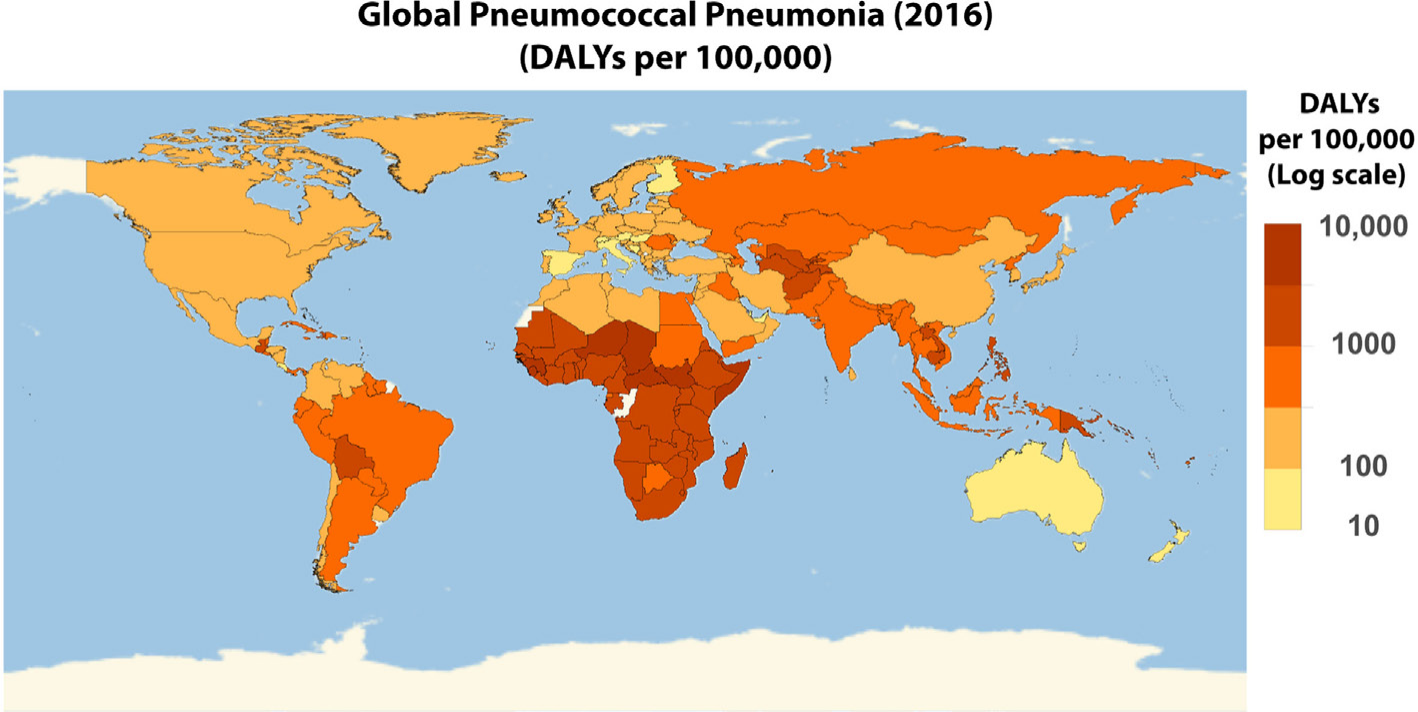
Worldwide disability adjusted life year (DALY) of pneumococcal pneumonia. Global distribution of pneumococcal pneumonia on a log10 scale of the 2016 DALY per 100,000 pneumococcal pneumonia data obtained from Institute for Health Metrics and Evaluation (7).

**Figure 6:**
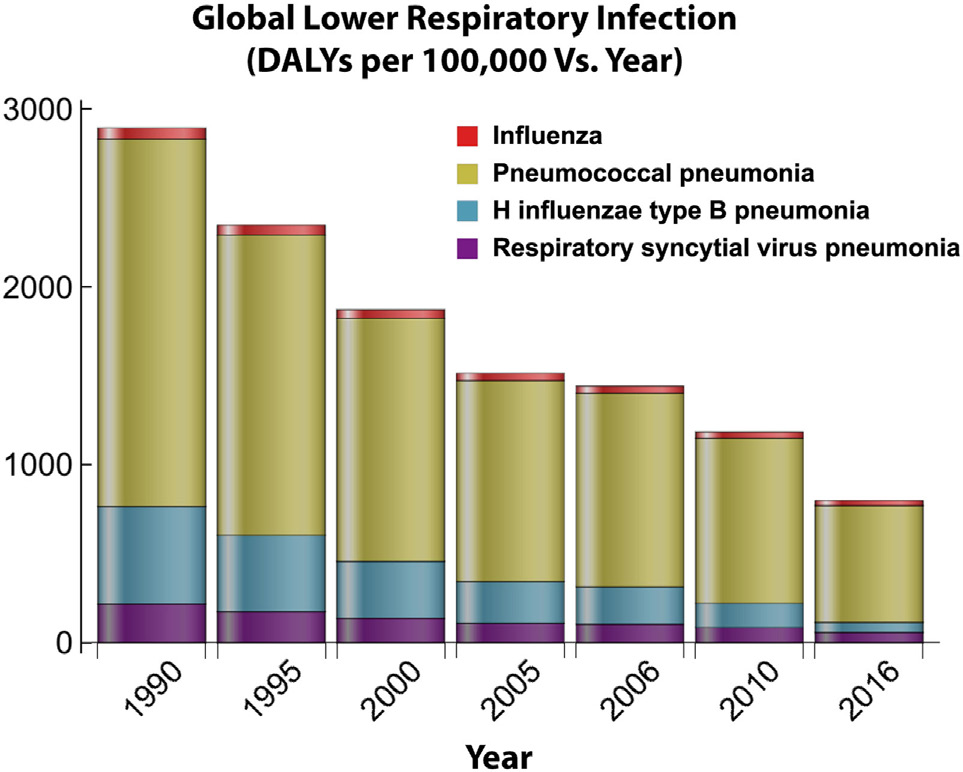
Global distribution of lower respiratory infections over time. This figure depicts how the burden for four major lower respiratory infections changes over time in response to the introduction of antibiotic treatments and vaccine implementation. Disability adjusted life year (DALY) data obtained from Institute for Health Metrics and Evaluation (7).

Pneumococcal pneumonia dominates as the main type of pneumococcal disease within the US and worldwide (24) (Figure 5). Overall, pneumonia is the eighth leading cause of death in the US (87), and is mainly caused by bacteria, but can also be caused by other pathogens such as viruses and fungi (22). For example, *Haemophilus influenzae* type b, respiratory syncytial virus (RSV), and influenza can also cause pneumonia, but pneumococcal pneumonia is the most prevalent (Figures 1, 2 and 6) (7). Over time the global disease burden of LRIs such as pneumonia has decreased, but they remain a healthcare concern for specific high-risk populations (Figures 2 and 6) (7). Worldwide pneumonia is the leading cause of death in children under the age of five (31, 88). The World Health Organization reported that a child dies from pneumonia every 20 s (89). There are approximately 900,000 cases of pneumococcal pneumonia (33, 90) that occur annually within the US (32, 90). In addition, United Nations Children Fund stated that in 2016, pneumonia accounted for 16% of the fatalities observed among young children under the age of five worldwide (92). Pneumococcal pneumonia leads to about 300,000–600,000 elderly hospitalizations annually in the US, and the elderly have reduced survival rates (93, 94). There are different types of pneumonia: community-acquired pneumonia (CAP), atypical pneumonia, hospital acquired pneumonia, and aspiration pneumonia (24). These differ based on where someone contracts the infection and what bacteria cause the disease. Currently, the most common form of pneumonia is CAP (which is mostly pneumococcal). This type of pneumonia spreads *via* person-to-person contact in the community, but outside of healthcare facilities, by breathing in aerosol droplets from a carrier or infected person (51, 95). Worldwide, CAP is currently the leading cause of death for young children who are under the age of five (29, 96). In 2015, 920,136 children died from CAP (97). Infants, young children, the elderly, smokers, and immunocompromised individuals are all at a higher risk of developing pneumonia due to a weakened immune system (22). CAP has a higher occurrence rate in the elderly compared to younger populations, and is also the fifth leading cause of death in the elderly population (93, 94).

### Transmission

The severity of pneumococcal diseases has led to multiple studies investigating how *S. pneumoniae* is transmitted. The nasopharynx has been classed as the main reservoir of *S. pneumoniae*. This is due to the nasopharynx of hosts being colonized without any symptoms (50). Following colonization, the spreading of the disease depends on carriers coming into close contact with healthy individuals within the community. The CDC has declared that the main source of *S. pneumoniae* transmission is direct contact with secretions of the respiratory system of a carrier (24). Le Polain de Waroux et al. (98) investigated transmission in 566 Ugandan subjects by studying nasopharyngeal samples, and determined that close interpersonal contact was necessary for the dissemination of *S. pneumoniae*.

Who exactly the main carriers/reservoirs of *S. pneumoniae* are, is still heavily debated. There have been a variety of studies trying to pinpoint which age group acts mainly as carriers/reservoirs for *S. pneumoniae* (99–101). Some researchers have suggested infants (9, 101), while others suggest that older children actually transmit the pathogen to infants (99, 100). Lipsitch et al.’s longitudinal study suggests that infants are reservoirs due to the duration of carriage and colonization (101). In this study, they also observed that the carriage time of *S. pneumoniae* decreases with age (99, 101). On the other hand, a longitudinal study investigating transmission and colonization in a daycare setting showed that toddlers act as a reservoir for *S. pneumoniae* and spread to family members (99, 100). Another contradicting study that used pre-existing data and mathematical modeling suggests that older children introduce the pathogen to their homes and transmit *S. pneumoniae* to younger children, siblings, and adults (99). Althouse et al. did confirm that there is higher colonization in infants, however, their results show that *S. pneumoniae’s* direction of transmission is instead from older siblings to infants as opposed to transmission from infants or parents to others in the household (99, 102). The duration of carriage seems to affect how well *S. pneumoniae* is transmitted as well as close contact between carriers and healthy individuals (98, 99). Althouse et al. concluded that despite the larger percentage of carriage being in infants, their role in transmission is minimal compared to that of toddlers and older children (99). The differences between these findings suggest that the direction of transmission is still not yet fully understood and further research is required. Another possibility would be that multiple age groups are acting as reservoirs rather than one specific group under different conditions.

In addition to close contact with an *S. pneumoniae* carrier, the bacterium may also be transferred to healthy individuals *via* fomites (103). Chronic carriers of *S. pneumoniae* can contaminate inanimate objects with biofilms (103). *S. pneumoniae* biofilms are able to survive being in the environment because the biofilm’s structure provides protection from drying out (47, 104). *S. pneumoniae* was found in high concentration on items within a daycare center following bacterial cultures (50, 103). Pneumococcus can survive being in the environment for long periods of time (for example, up to 4 weeks) (103, 105). Because of this, fomites can serve as a reservoir. These findings indicate why it is important to improve hygiene and cleanliness in everyday-life, and at community-based facilities and daycare centers.

*Streptococcus pneumoniae* also makes a toxin, pneumolysin, that promotes shedding and in turn enhances bacterial transmission (59). Pneumolysin induces inflammation in hosts during colonization and this promotes bacterial shedding (59). Zafar et al. conducted a shedding assay which suggests that *S. pneumoniae* may be using the host’s inflammatory response as a signal for initiating its exit from the inhospitable host (59).

#### Transmission *via* Coinfections

Coinfection with *S. pneumoniae* is often seen during viral infections such as influenza, also the eighth cause of death within the US (87), and RSV. Coinfections by pathogenic bacteria such as *S. pneumoniae* increase the severity and mortality rates of viral infections (106, 107). For example, during the influenza pandemic of 1918, the analysis of lung samples from those infected indicated that a majority of the deaths were due to bacterial infections and not the influenza virus (107–109). Coinfection is possible due to the pre-existing damage on the epithelia of the respiratory tract which promotes bacterial colonization (110–113). More specifically, *S. pneumoniae’*s bacterial load increases during viral coinfections due to the bacteria’s attachment to cells that are already infected by the virus (114). Studies have also shown that colonization of *S. pneumoniae* is affected by flu vaccines, which also indicates that *S. pneumoniae* benefits from colonizing hosts that are already compromised (107, 115). Increased host colonization and bacterial cell density of *S. pneumoniae* during viral infections promote transmission (115). Khan et al. determined that there are higher risks of bacteremia, mortality, and spread to other tissues during coinfections (110, 115). Co-detection with *S. pneumoniae* has also been observed in RSV infections (116).

## Virulence Factors

*Streptococcus pneumoniae*, like many other bacterial species, produces toxins that are harmful to its host, has several surface proteins and physical structures, which play a vital role in its pathogenesis (27). These virulence factors (Figure 4; Table 2) work by hindering the host’s immune system response, avoiding defense mechanisms, or by direct contact with host tissues and surface receptors, which in turn interferes with the host’s immune system activation and bacterial clearance (27). As discussed above, *S. pneumoniae* exploits hosts with weakened or compromised immune systems (13–15). *S. pneumoniae’s* effectiveness in causing infections is directly related to the host immune system’s developmental stage and possible deterioration with aging (see also Section “Host Immune System Responses to *S. pneumoniae*”).

*Streptococcus pneumoniae’s* virulence thrives because of the bacteria’s ability to acquire new genetic material *via* transformation and recombination (117). Investigating the level of genetic variation within *S. pneumoniae* is important for not only thoroughly understanding its virulence but also for developing effective treatments and vaccines. About 4,000 *S. pneumoniae* genomes have already been sequenced (117), with lengths ~2–2.2 million bp (54). More than 2,000 genes have been annotated, but novel genes are still regularly discovered as more sequences become available (117). Variation in gene content and single genes plays a role in defining the virulence profile of some of *S. pneumoniae* strains (117). Donati et al. describe genome diversification as *S. pneumoniae’s* ability to evolve in diverse host environments (117, 118). Genetic variation has been observed within identical *S. pneumoniae* clones, due to changes in gene content of their dispensable genes (117, 119). Dispensable genes are not needed for bacterial growth (117), but provide selective advantages to *S. pneumoniae* such as antibiotic resistance (120). Additional variants are introduced to the core genome of *S. pneumoniae via* allele replacement. This is because the bacteria lacks SOS genes and does not repair damaged DNA (121). Carriage can also influence genetic variation. In 2017, Lees et al. developed a model to assess carriage duration and assembled those findings with data from whole genome sequencing. The results indicated that pneumococcal genetic variation accounts for the phenotypic variation compared to host’s age and previous carriage (5%) (122).

The major virulence factors of *S. pneumoniae* that have been thoroughly characterized are summarized in Table 2. Below we further discuss virulence factors of particular interest:

1. *Polysaccharide capsule: S. pneumoniae’s* extracellular polysaccharide capsule, the most important virulence factor (55), helps to initiate infection by allowing the bacterium to adhere to host cells and cause inflammation, while also providing protection from the host’s immune system (54, 55). The capsule inhibits phagocytosis by innate immune cells, prevents the recognition of the bacterium by host receptors and complement factors, and also avoids neutrophil traps (17, 27, 55, 56, 71, 123). Many serotypes of *S. pneumoniae* are characterized by the polysaccharides that are on the outer coat of the capsule, and they are all pathogenic in their own unique manner—some more harmful than others (20, 56). For example, serotype 1 has been found in invasive infections which have lower fatalities, whereas serotype 3 is associated with colonization of the nasopharynx and serious infections which can lead to fatalities (39, 42, 56, 124, 125). The capsule manipulates how immunoglobulins recognize the bacteria (126) and inhibits the host’s defenses such as mucus layers and cilia from removing the bacterium, and is vital for pneumococcal bacterial cells’ colonization (57). The roles of the capsule in pathogenesis have been described to be due to its charge (57, 127). The capsule has a negative net charge which is in part due to the acidic polysaccharides and phosphates that make up this layer (57, 127). The charge is important because it defines how interactions with other cells take place, specifically host cells (57, 127). One explanation for *S. pneumoniae’s* ability to avoid being trapped by mucus layers and phagocytic cells is due to electrostatic repulsion (57, 127). Negatively charged mucus and phagocytic cells, such as macrophages, have led to a reduction in the clearance of *S. pneumoniae* because of this electrostatic interaction (57, 127).
  *Streptococcus pneumoniae’s* virulence *via* its polysaccharide capsule is enhanced by its ability to undergo capsule switching (117, 128, 129). Mutations in the capsule polysaccharide synthesis genes (*cps*) promote serotype switching (117, 128, 129). Serotype switching in strains is increasingly being observed and it is often *via* recombination or polymorphisms based on antibiotic and vaccine selective pressures (further discussed in Section “Prevention, Antibiotic Response, and Age-Dependent Immune Responses”) (117, 128, 129). Currently, serotype switching is a healthcare concern as non-vaccine serotypes are being detected at higher rates compared to before vaccines were implemented (128). Moreover, mutations in novel genes or a disruption of the *cps* loci can lead to *S. pneumoniae* strains without capsules (130). Non-typeable *S. pneumoniae* cannot effectively colonize hosts, but novel genes such as pneumococcal surface protein K in the *cps* loci assist with adhesion (130). Serotype switching and capsule-free strains of *S. pneumoniae* together will add to the burden on the high-risk age groups (infants and the elderly) (130), and because of this vaccines and treatments should be improved.
2. *S. pneumoniae’s cell wall components: S. pneumoniae* is a Gram-positive bacterium with a thick cell wall. The cell wall is important because it provides protection and shapes the cell (131). Peptidoglycan, wall teichoic (WTA), and lipoteichoic acids (LTAs) are the main components of *S. pneumoniae’s* cell wall (131). WTAs are covalently attached to peptidoglycan whereas LTAs are non-covalently connected to the cytoplasmic membrane with a lipid anchor (131). The capsular and cell-surface proteins are all linked to the peptidoglycan (131). Alternating glycan chains of *N*-acetylglucosamine (GlcNac) and *N*-acetylmuramic (MurNac) acids crosslinked by peptides make up peptidoglycan (131, 132). These glycan chains can undergo secondary modifications such as deacetylation of GlcNac and O-acetylation of MurNac (131, 132). These modifications aid in *S. pneumoniae’s* virulence by making the cell resistant to lysozyme (132). Cell wall components, WTA, and LTA have phosphorylcholine (PCho) residues which serve as anchors for choline-binding proteins (CBPs). CBPs are important for host–pathogen interactions such as evasion of host immune responses (discussed later in this section) (131, 132). PCho in bacterial cells is unusual and *S. pneumoniae* are currently the only bacteria known to require it for growth (132). WTA, LTA, and peptidoglycan are pathogen-associated molecular patterns (PAMPs) that can cause an inflammatory response in hosts. Peptide synthesis, peptidoglycan structure, WTA, and LTA synthesis and modifications have been further discussed by Gisch et al. (131).
3. *Pneumolysin*: this toxin that is capable of forming pores in cell membranes (133) can be found in the cytoplasm of *S. pneumoniae* and other Gram-positive bacteria (27, 60, 133). Pneumolysin is released as a result of cell lysis and is toxic to host cells (27, 61, 134). Pneumolysin binds to membranes containing cholesterol (135), and forms pores which later lead to host cell lysis (27, 56, 62). In addition to causing cell lysis, pneumolysin plays a role in promoting the formation of biofilms (63), it reduces mucus clearance of the bacterium, and it can interfere with the host’s immune system (27, 60, 61, 134, 136). Pneumolysin regulates the complement system (54) and reduces phagocytosis by innate immune cells. It is also a pro-inflammatory toxin which causes damage to host cells. It can regulate cytokine and chemokine production (22, 51). This pro-inflammation has also been shown to assist with host-to-host transmission (59). By increasing cell inflammation, there is an increase in shedding and thus a higher rate of transmission of the bacteria (59).
  Studies have also shown that pneumolysin can cause DNA damage by inducing double-stranded DNA breaks. One mechanism of DNA damage by pneumolysin was described by Rai et al. in 2016 (64). They showed that the toxin can dysregulate the production of reactive oxygen species (ROS) intracellularly (64). This is possible because of pneumolysin’s pore-forming properties—it creates ion channels that disrupt cell calcium levels, which leads to overproduction of ROS, that then causes DNA damage (64). Host DNA damage may lead to increased pneumolysin virulence in the elderly, who are already experiencing a compilation of DNA damage and telomere shortening due to aging (137).
  Pneumolysin has different allelic forms that and can also affect the toxin’s hemolytic activity (54, 138). For example, genetic variation in allele 5 produces a non-hemolytic form of pneumolysin (138–141). Previously, a cysteine residue at amino acid position 428 was identified in the conserved sequence described to be important for the hemolytic activity of pneumolysin (142). However, cysteine was later substituted by alanine without affecting the toxin’s hemolytic activity (138, 143).
4. *Autolysin*: this enzyme is involved in autolysis of bacteria which results in the release of pneumolysin, teichoic acid, and other components from within the cell (22, 51). An example of this is lytic amidase (LytA) (67), a choline-binding amidase (17) (see below) that degrades peptidoglycan and causes cell lysis (27, 68, 69, 144). Autolysins promote colonization of nasopharyngeal cells due to the release of toxins such as pneumolysin during cell wall degradation (27).
5. *Pneumococcal surface proteins: S. pneumoniae* has a large variety of surface-exposed proteins (17, 72) that aid in its pathogenesis by acting as adhesins to host cells and hindering the host’s immune system, specifically the complement system (22, 27, 51, 145, 146). Pneumococcal surface proteins are categorized into four groups: CBPs, lipoproteins, non-classical proteins, and proteins that have an LPXTG motif (X represents any amino acid) and can be covalently bound through sortase cleavage of the motif (17, 72).
  - *CBPs: many* of *S. pneumoniae’s* surface proteins are classed as CBPs. For example, pneumococcal surface proteins (discussed below) are also classified as CBPs (27, 72, 76, 77). These proteins are known for binding to PCho on *S. pneumoniae’s* cell wall (27, 72, 76, 147) and are necessary for adhesion to host cells (27, 76, 147). CBPs affect the host’s complement system by blocking its activation and reducing the ability of immunoglobulins to eliminate the pathogen (27, 72). Some of these CBPs can also modify host cell surfaces to allow for binding interactions between to host cell receptors and *S. pneumoniae* (76). *S. pneumoniae* has approximately 10–16 identified CBPs (17, 148–150) including pneumococcal surface protein A (PspA), pneumococcal surface protein C (PspC), and LytA which are discussed below:
    - *PspA* is very electronegative, and this characteristic can block complement binding, which prevents opsonization of *S. pneumoniae* (17, 73). PspA can also bind to host lactoferrin (22, 27, 51), specifically apolactoferrin (iron-free), which in turn provides protection to *S. pneumoniae* against the bactericidal killing of apolactoferrin (151, 152).
    - *PspC*, also known as CbpA (highly polymorphic), promotes adherence by binding to the polymeric immunoglobulin receptor (72, 153). It facilitates the colonization of *S. pneumoniae* into the nasopharynx and can prevent the formation of C3b (part of the complement system) by binding to factor H. This in turn interferes with opsonization of *S. pneumoniae* (17, 72, 154). PspC exists in multiple allelic forms with most alleles containing a C-terminal cell wall choline-binding motif. However, there are also 17 allelic variants that have the LPTXG motif (see LPXTG cell wall bound proteins) (54, 155, 156). In addition, allelic variant PspC 4.4 was characterized as a ligand for complement inhibitor C4b-binding protein (54, 157), which leads to an allele-dependent form of protection from the complement (157).
    - *LytA*, an autolysin, was the first of three major lytic enzymes found in *S. pneumoniae* (76, 158). LytA degrades peptidoglycan by cleaving the *N*-acetyl-muramoyl-l-alanine bond (72, 159). This causes cell lysis and the release of pneumococcal antigens such as pneumolysin, peptidoglycan, and teichoic acids which are all harmful to host cells (72, 159, 160). The release of these harmful particles from *S. pneumoniae* cells is also capable of inhibiting cytokine [such as interleukin (IL)-12] production, which in turn blocks the activation of phagocytes (158, 161). This is thought to be due to the fact that cells are already decomposed so phagocytic activity is no longer necessary (158), and acts as a form of immune system evasion by *S. pneumoniae* (161–163). By blocking cell signaling *via* cytokine production, LytA has also been shown to hinder complement activation (76). How exactly this blockade might be happening, still needs to be further researched.
    - *LytB and LytC* are two other lytic enzymes found in *S. pneumoniae*. Their roles in *S. pneumoniae’s* virulence are not as thoroughly understood as LytA. Studies have shown that LytB is necessary for separating daughter cells (164, 165). LytC on the other hand, is described as a lysozyme. Ramos-Sevillano et al. have indicated that LytB and LytC interact and are both involved in adhesion of *S. pneumoniae* to epithelial cells within the nasopharynx of hosts. Their results also suggest that LytC helps with evasion of the complement system *via* experiments with mutants. LytC mutants had larger amounts of C3b deposition and LytB and LytC double mutants all had a reduction in their ability to adhere to host cells (166). These findings shed light on the roles of LytB and LytC. They aid in virulence by playing a role in colonization and evasion of host immune responses (166, 167). In addition, LytC has also been described to play a role in cellular fratricide with LytA. These enzymes are released to lyse non-competent pneumococci in close proximity of competent cells (168). This is important for transformation of *S. pneumoniae*. Competent cells are able to uptake and incorporate free DNA from the lysed cells (168, 169). This promotes genetic exchange which in turn can improve bacterial survival. For example, the bacterium can take up genes for antibiotic resistance (168, 169). LytC’s activity is most active at 30°C which indicates it is probably most active in the upper respiratory tract (168, 169).
    - *CbpF*, the most abundant protein on *S. pneumoniae’s* cell wall is capable of regulating LytC (77, 170). CbpF regulates LytC’s activity by blocking LytC’s access to its substrate (77, 150, 170).
    - *Other CBP*: there are about eight other CBPs: CbpD, CbpG, CbpI, CbpJ, CbpK, CbpL, CbpM, and CbpN. These have not been studied as extensively as the main CBPs previously discussed. There is not much known about their structure or function. CbpD has been shown to be involved in fratricide by working with LytA and LytC (150, 169, 171). The CbpD is able to provide a substrate for LytC that is more accessible by binding to target cells and breaking down the cross-links of the peptidoglycan (150). CbpG is necessary for adhesion and all others been reported to work as adhesins (77, 150).
  - *Lipoproteins: these* proteins are necessary for substrate transport. There are approximately 50 lipoproteins that have been characterized (72, 148, 150). The four main lipoproteins are the pneumococcal surface adhesin A (PsaA), pneumococcal iron acquisition A (PiaA), pneumococcal iron uptake A (PiuA), and pneumococcal iron transporter (PitA) (17, 72, 172, 173). They are all metal-binding proteins that combine with ATP-binding cassette (ABC) transporter complexes. ABC transporters transport substrates across membranes by utilizing energy generated from ATP binding and hydrolysis.
    - *PsaA* is involved in transporting magnesium and zinc into the cell (27, 74, 174, 175). Investigations have previously reported PsaA’s role in cell adhesion and promoting cell invasion of *S. pneumoniae* (74, 176). However, other studies on PsaA mutants have found that PsaA has no clear role in adhesion, but rather magnesium transport (175). This particular characteristic of adhesion needs to be further investigated (26). Also, genetic mutations can alter PsaA’s function which may lead to impaired ability to acquire manganese which results in decreased resistance to oxidative stress (54).
    - *PiaA, PiuA, and PitA* are involved in regulating iron-uptake (177, 178). In addition to this, PiaA and PiuA have been described to be needed for full virulence of *S. pneumoniae* in mice (177, 179). Mutations in PiaA and PiuA affect growth and virulence of *S. pneumoniae* (180, 181). This indicates the importance of iron in the environment for growth. Furthermore, Cheng et al. in 2013 crystalized PiaA and discovered that PiaA is capable of binding to ferrichrome (182–184) despite previous findings suggesting pneumococci do not produce siderophores (185). Cheng et al. concluded that *S. pneumoniae* is probably able to acquire holo-siderophores from other bacteria within the host (181, 182). On the other hand, PiuA binds to both hemin and hemoglobin but has greater affinity for hemin (183, 186). PitA was later discovered and characterized to bind to ferric irons (172, 173, 183). A novel iron transport was discovered in 2016 by Yang et al. (187) *via* proteomics. In this study, they constructed a triple mutant by deleting PiaA, PiuA, and PitA (187). Using this mutant, they were able to identify potential iron transporters, such as putative protein SPD-1609, which functions similarly to PitA *via* translatomics and proteomics (187). These findings suggest that there are potentially more iron-binding proteins in *S. pneumoniae* to be discovered and that the bacteria have developed transport systems to ensure they have access to as much iron as possible for their survival.
  - *LPXTG cell wall bound proteins* are recognized by the sortase of the cell wall (54, 149, 188). Sortase recognizes the LPXTG sequence, cleaves at this site, and anchors the proteins to the cell wall (54, 188, 189). Mutating the sortase gene srtA caused a decrease in *S. pneumoniae’s* adhesion to host nasopharyngeal cells *in vitro*, and caused neuraminidase to be released from the cell well into the media (190). Neuraminidase is an example of an LPXTG cell wall bound protein and is known for cleaving sialic acid from glycoproteins. In the case of pathogenesis of *S. pneumoniae*, this activity can lead to the removal of sialic acid from lactoferrin which hinders lactoferrin’s bactericidal effect. Neuraminidase is secreted from *S. pneumoniae* cells and targets host cells (54, 188). It is also involved in colonization of the host and has been suggested to be involved with adhesion (150, 190).
  - *Non-classical surface proteins (NCSPs)* are found on *S. pneumoniae’s* surface, but do not have a membrane-anchoring motif nor a leader peptide (72). They are also known as moonlighting proteins for having multiple functions (72, 78, 148). NCSPs function as adhesins that are able to bind to host molecules which promotes pneumococcal host cell invasion (148). There are two main NCSPs: pneumococcal adherence and virulence factor A (PavA) and glycolytic enzymes [enolase and glyceraldehyde 3-phosphate dehydrogenase (GAPDH)] (150).
    - *PavA* attaches to fibronectin and assists with adherence to host cells (149). PavA also provides protection to pneumococci by controlling inflammation and inhibiting recognition by dendritic cells (191). PavA mutants were more susceptible to recognition and phagocytosis by dendritic cells compared to wild type (191). In addition to this, when the dendritic cells encountered PavA mutants there was a reduction in cytokine production, which affected the adaptive immune response. These findings characterize PavA’s potential function in immune system evasion by *S. pneumoniae* and cytokine production by dendritic cells (191).
    - *Enolase and GAPDH* are both plasminogen-binding proteins. Enolase is an anchorless protein found at the surface of *S. pneumoniae* (192). It is important for proteolytic activity on the cell surface (193), which is necessary for the pathogenesis of *S. pneumoniae* (192). Enolase also promotes complement system evasion by binding to the complement inhibitor C4b-binding protein (194). In addition, studies suggest that enolase may cause host tissue damage by inducing the production of neutrophil extracellular traps by binding to neutrophils (195). GAPDH can be found on the surface and in the cytoplasm of *S. pneumoniae* (196). Although GAPDH binds to plasminogen, it has a higher affinity for plasmin (149, 196). GADPH is suggested to also play a role in iron acquisition due to its ability to bind to hemoglobin and heme (149). Like enolase, GADPH may also play a role in host cell invasion and evasion of the immune system. LytA has recently been identified to be involved in the delivery of GADPH to *S. pneumoniae’s* cell surface (79).
6. *Pili*: these hair-like structures are located on the cell surface of *S. pneumoniae* and many other bacteria (27, 51, 81). They assist with *S. pneumoniae’s* attachment and colonization of epithelial cells within the nasopharynx and lungs of hosts (51, 81, 82). These pili also help the bacteria avoid phagocytosis by host immune cells (22). There are two main types of pili found on *S. pneumoniae*: pilus-1 and pilus-2. Pilus-1 is found in 30% of clinical isolates (197) whereas pilus-2 is only in about 16% (198). Studies have shown that piliated *S. pneumoniae* induce higher tumor necrosis factor (TNF) responses than the non-piliated during pneumococcal infection (81). This suggests that pili are able to stimulate inflammatory responses of the host (81). Pancotto et al.’s findings indicated that pilus-1’s expression is regulated *in vivo* (82). High expression of pilus-1 is observed at early stages of colonization and reduced expression during later stages of infection. This downregulation may be necessary for avoiding host immune response but this needs to be further investigated as it is not clear why this might be happening (82). *S. pneumoniae*, like many other pathogenic bacteria have a type IV pilus that is necessary for transformation (199, 200). This pilus is formed on the surface of the bacterial cell and contains the major pilin ComGC. The operon that codes for ComGC also encodes for an ATPase which is needed for powering the pilus assembly. The structure of ComGC was recently discovered by Muschiol et al. in 2017 (199).
7. *Immunoglobulin A1 (IgA1) protease*: this enzyme is produced by *S. pneumoniae* and it works by cleaving the human IgA1 into fragments (83, 84). The IgA1 represents an isotype of immunoglobulin A (IgA) which has two isotypes: IgA1 and IgA2 (201). These two isotypes differ in hinge regions—IgA1 has an extended hinge region because of an insertion into this region of a set of duplicated amino acids (201). IgA1 proteases reduce the binding IgA’s effector region of the heavy chain and hinder killing of the bacterium by these antibodies (83, 85).
8. *Hydrogen peroxide: S. pneumoniae* secretes hydrogen peroxide (H_2_O_2_) which causes damage to host DNA (202). However, this is only observed in strains with pyruvate oxidase activity (SpxB gene) (202, 203). H_2_O_2_ production also has bactericidal effects. *S. pneumoniae* uses this to reduce the growth of bacteria it may be competing with (203). In addition, pneumococcal H_2_O_2_ induces an innate immune response by enhancing the release of pro-inflammatory cytokines, and targets cellular stress responses (204). As *S. pneumoniae* produces H_2_O_2_ *via* pyruvate oxidase, hydroxyl radicals form *via* the Fenton reaction (205). These radicals are often harmful to bacteria but do not affect *S. pneumoniae*. This is because of *S. pneumoniae’s* ability to reduce reactive OH before it comes into contact with DNA (206), by sequestering Fe^2+^ away from DNA (206). In addition to producing H_2_O_2_, the *SpxB* gene has also been found to increase resistance to H_2_O_2_ (207). *SpxB* mutants produced no H_2_O_2_ and were less resistant (207). In addition, *S. pneumoniae* has a variety of defense proteins involved in detoxification, repair, regulation, and cation homeostasis that provide protection against oxidative stress (206).
9. *Pathogenicity islands (PAIs)*: these are parts of pathogenic bacterial genomes that were acquired *via* horizontal gene transfer (208). The genes on PAIs aid in the virulence of the pathogen (209). PAIs can code for iron-uptake systems and proteins involved in cell attachment (209). For example, the first PAI discovered in *S. pneumoniae*, pneumococcal PAI 1 codes for the PiaA iron transporter complex (180). In addition, the pilus-1 is encoded by another PAI, known as the rlrA islet (81). However, this PAI is not found in all of the *S. pneumoniae* clinical isolates (81). Pilus-2 is also encoded by a PAI, pilus islet 2 (198). Another important adhesin, pneumococcal serine-rich repeat protein (PsrP), is also coded for by a PAI. PsrP is important for *S. pneumoniae’s* attachment to cells within the lungs (210). High PsrP production is also linked to biofilm growth (211). PAIs promote genetic variation in species, and this may affect current treatment and vaccine targets.
10. *Biofilms*: these are structured communities that consist of aggregated microbial cells surrounded by an extracellular matrix of polysaccharides that attach to surfaces (47, 212). The extracellular matrix provides protection and enhances *S. pneumoniae’s* virulence (47, 212). Biofilms are formed in response to stress and harsh conditions to promote bacterial survival (47, 212, 213). To promote biofilm formation and competence, *S. pneumoniae* downregulates expression of capsular proteins (214). Within biofilms, horizontal gene transfer rates increase due to close cell proximity (47, 212, 215, 216). Studies indicate that *S. pneumoniae* biofilms are not effectively cleared during antimicrobial treatments due to increased antimicrobial resistance (217). In addition, *S. pneumoniae* biofilms are able to escape host immune responses such as mucociliary clearance (218).

## Host Immune System Responses to *S. pneumoniae*

**Figure 7:**
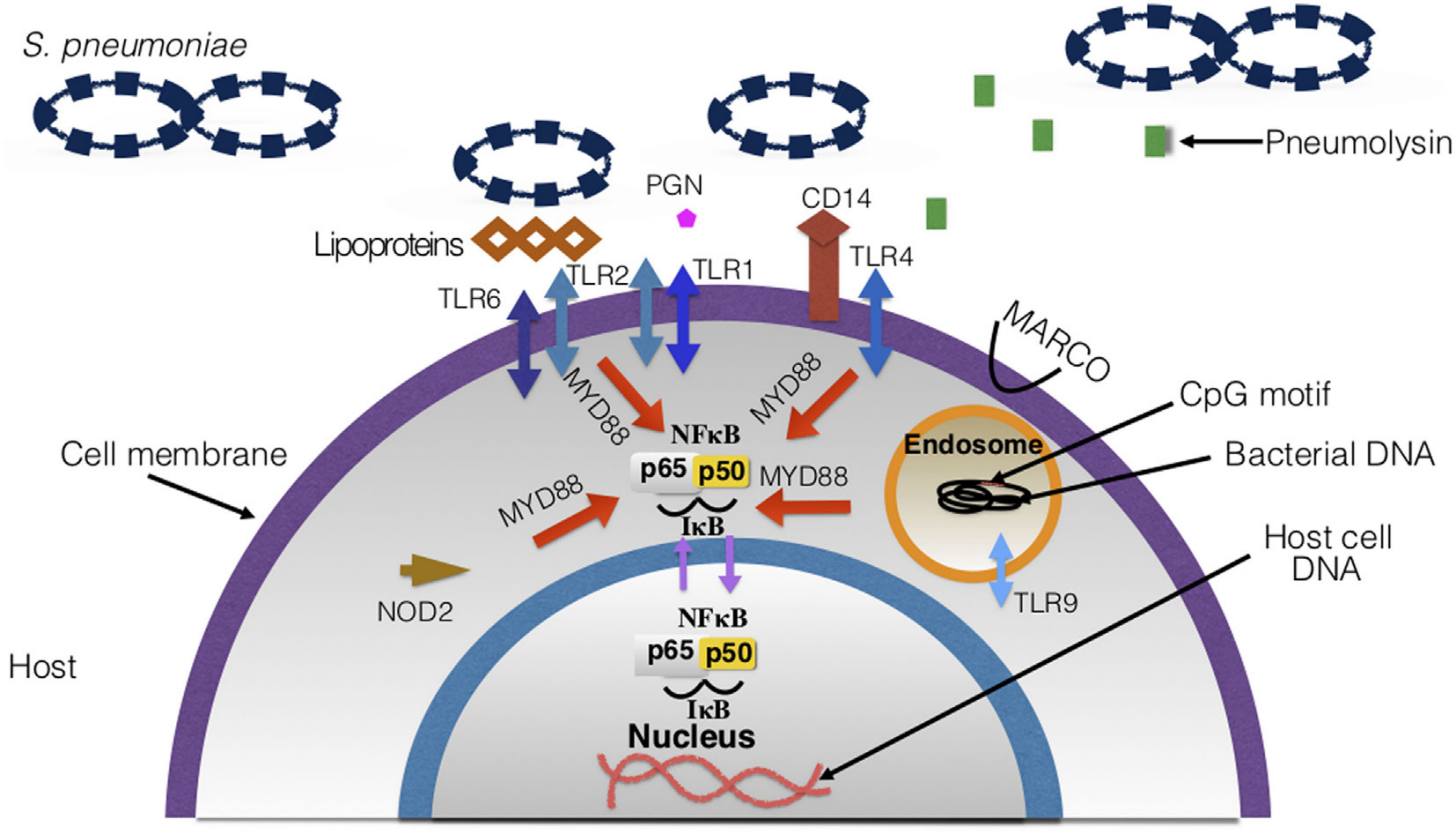
Host surface and intracellular receptors necessary for immune response to *Streptococcus pneumoniae*. Highlighted in this figure are the major pathogen recognition receptors necessary for binding to pneumococcal ligands and eliciting an immune response. Upon binding to the ligands, receptors and signaling pathways are activated, which leads to the overall production of inflammatory cytokines and recruitment of immune cells. There are 10 toll-like receptors (TLRs) that have been discovered in humans—TLRs involved in pneumococcal disease are depicted in the figure.

We have discussed above the virulence factors that aid in ensuring *S. pneumoniae* can evade the host’s immune system. On the other hand, there are several host defenses that recognize *S. pneumoniae*, act rapidly, and clear the pathogen before it can cause pneumococcal diseases. Protection from *S. pneumoniae* is dependent on the state of the host’s immune system. Age plays a role in how successful the immune system will be at clearing the infection by *S. pneumoniae*. Children under the age of five and the elderly are at higher risk for contracting pneumococcal diseases (Figure 2). This is due to infants having a naïve immune system, whereas the elderly are experiencing immunosenescence (28). A variety of immune cells are involved in the innate (first-line of defense) and adaptive immune responses. The most important immune cellular and humoral components for defending against pneumococcal infections (Figure 7) are summarized in the following sections including how aging may affect their ability to defend the host.

### Innate Immune Responses

**Figure 8:**
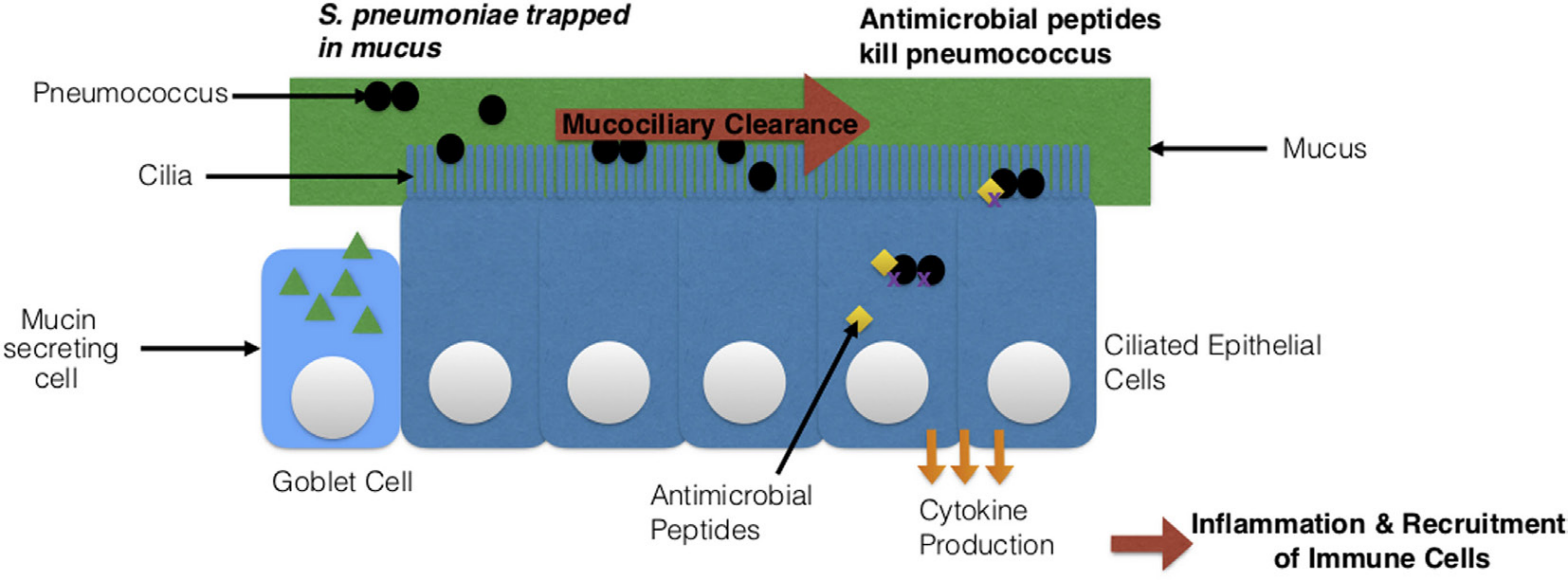
*Streptococcus pneumoniae’s* interaction with host epithelial cells. Two types of epithelial cells are depicted: goblet cells and ciliated epithelial cells. The cilia on the epithelial cells together with the mucus produced by goblet cells clear the pathogen *via* mucociliary clearance. Epithelial cells can also secrete antimicrobial peptides that directly kill *S. pneumoniae* or produce cytokines, which leads to a state of inflammation and the recruitment of immune cells.

**Figure 9:**
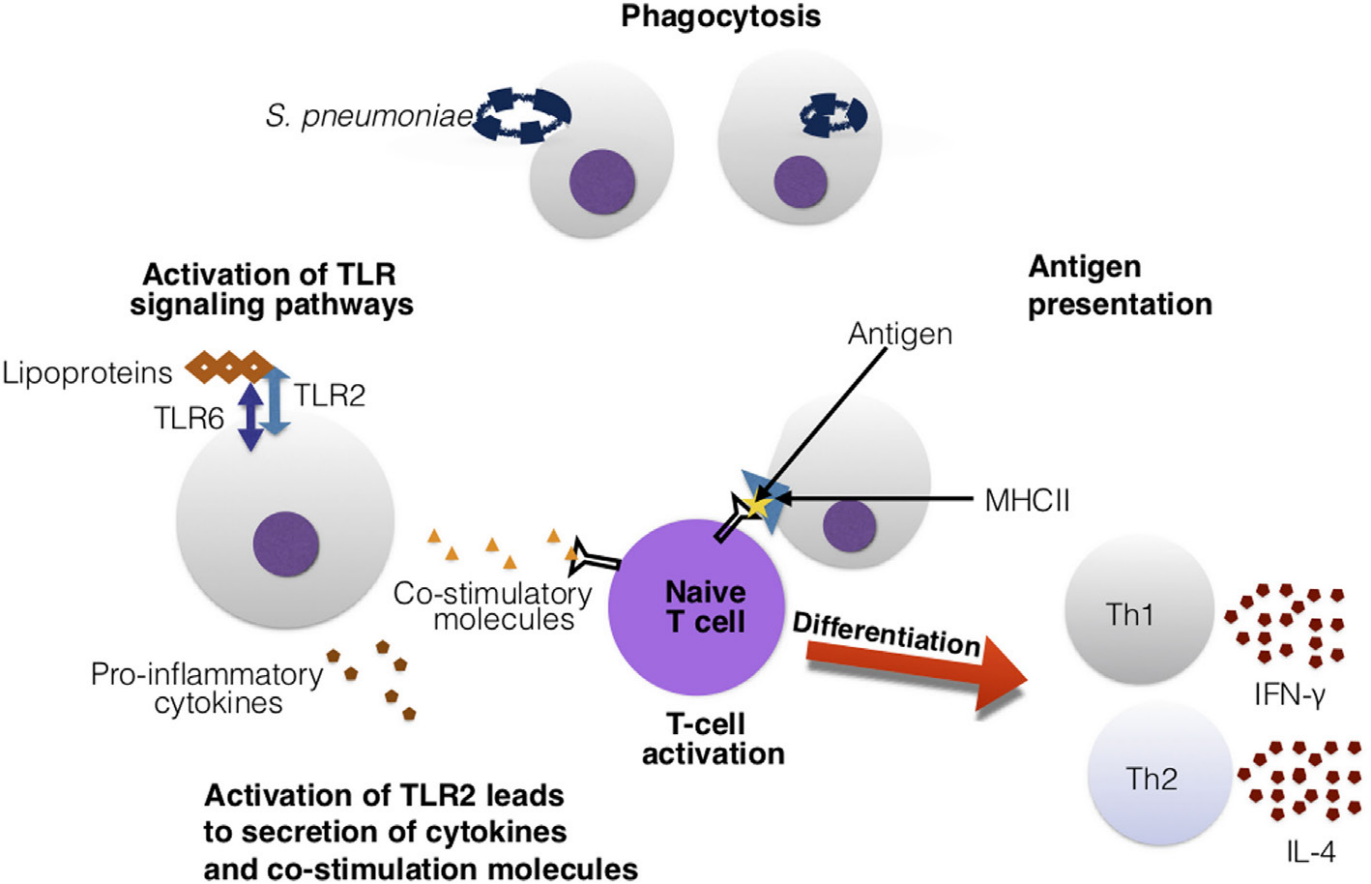
Toll-like receptors (TLRs) assist in the activation of adaptive immune cells. In this figure, TLR2 recognizes the *Streptococcus pneumoniae’s* lipoproteins. Upon activation, TLR2 secretes cytokines and co-stimulatory molecules. These co-stimulatory molecules are essential for co-stimulation and activation of T cells. The T cell is presented an antigen with major histocompatibility complex (MHC)II and antigen-presenting cell. The recognition of the antigen–MHCII complex and the co-stimulatory molecules activates the T cell and leads downstream to differentiation into Th1 and Th2 cells, that can release various cytokines such as interferon-gamma (IFN)-γ and interleukin (IL)-4.

*Innate immunity* involves non-specific immune responses—cells and receptors recognize foreign particles and elicit immune responses to eliminate the invaders that can be harmful to the host (16, 159, 219). Cell-related innate immune responses against pneumococcal infection include:

- *Mucosa and respiratory epithelial cells*: epithelial cells provide a protective barrier for tissues and organs (219). In this case, they line the respiratory tract and protect against pneumococcus (219). There are epithelial cells known as goblet cells, which secrete mucus (220). The negatively charged mucus is necessary for maintaining moisture and trapping foreign particles and pathogens. In addition, ciliated epithelial cells function simultaneously with the mucus to clear pathogens. This process is known as mucociliary clearance (57). Once the pathogen is trapped in the mucus, the cilia (hair-like structures) move together to direct the trapped pathogen and the mucus to the mouth, for expelling the pathogen *via* coughing or swallowing (221). The respiratory epithelial cells can recruit other cells by producing and releasing cytokines and chemokines (22, 220). They also can directly kill pneumococcus by secreting antimicrobial peptides such as defensins, human apolactoferrin, and lysozyme (Figure 8) (22, 220, 222). Human apolactoferrin sequesters iron and lyses cells. Lysozyme also lyses cells and acts as a bactericidal (222). d-alanine in teichoic acids of *S. pneumoniae’s* cell wall help to evade killing by antimicrobial proteins (positively charged) by reducing the negative charge (223, 224). The negatively charged capsule also promotes evasion of mucus *via* electrostatic repulsion (225). Mouse model experiments showed that encapsulated *S. pneumoniae* were easily trapped in mucus and unable to migrate to the epithelial cells when compared to capsulated *S. pneumoniae* (57). This again was due to the negatively charged capsule. In addition to this, *S. pneumoniae*’s neuraminidase degrades mucin and reduces the negative charge by removing sialic acids (225–227). As previously mentioned in Section “Virulence Factors,” the structure of peptidoglycan can be modified. This modification promotes resistance of *S. pneumoniae* to lysis *via* the lysozyme (228). Another impressive method of evasion by *S. pneumoniae* is its ability to undergo phase variation (229, 230). *S. pneumoniae* is able to express a thick and a thin capsule under certain conditions (229, 230). The thick capsule is necessary to avoid entrapment in mucus, and the thin capsule is necessary for binding directly to epithelial cells (229, 230). Once the thin capsule is expressed, adhesins are exposed for binding to the glycoconjugates on epithelial cells (229, 230).
  Infants and the elderly both are challenged with mucociliary clearance due to different reasons: in infants, immature submucosal glands, surface epithelial secretory cells, and low numbers of ciliated epithelial cells can result in poor mucociliary clearance (231). In the elderly, as the host ages there is a deterioration of mucociliary clearance, with reduced mucin and slower cilia beat frequencies (28, 232), which promotes dissemination of the bacteria (28). As *S. pneumoniae* virulence factors can also degrade mucus and slow down cilia (28), immaturity and deterioration of mucociliary clearance could cause disease exacerbation through increased colonization and recurrent infections.
- *Phagocytes*:
  - *Neutrophils*: these are found in larger concentrations compared to any other white blood cells (WBC), and they are generally the first to travel to the infection (233, 234). Neutrophils are phagocytic cells (234) that also produce granules, which break down the cell walls of pathogens ultimately killing them (234). There are two main types of granules produced by neutrophils: primary and secondary, which differ based on age/maturity of the neutrophil (219, 235). Primary granules include defensins whereas secondary granules include enzymes necessary for digestion, such as lysosomes. Neutrophils can also trap *S. pneumoniae* extracellularly, by using extracellular fibers made up of DNA (236).
    Neutrophil response changes with age: infants experience minimal protection by neutrophils in their early days of life due to poor bactericidal function, impaired phagocytotic activity, low response to inflammatory signals, and reduced chemotaxis (237–239). With age, neutrophil activity improves and strengthens in young adults but later starts to deteriorate. Elderly populations experience impaired chemotaxis, which may lead to the overproduction of proteases by neutrophils. This causes an increase in inflammation levels in older subjects (15, 240). Neutrophil extracellular traps generation, phagocytosis, and killing diminishes with age (15, 241).
  - *Macrophages*: macrophages are derived from monocytes (219) and function as phagocytic cells that engulf and directly kill *S. pneumoniae* (16, 219). These cells can recruit other immune cells, such as neutrophils *via* cytokine signaling (242), and remove dead neutrophils (159, 243) and other cells *via* phagocytosis and apoptosis. Macrophages attack cells that have been opsonized by the complement system and Fcγ receptors (225). The macrophage receptor with collagenous structure (MARCO) (244), found on the surface of macrophages, aids with the phagocytosis of non-opsonized antigens (244). Macrophage activation due to *S. pneumoniae’s* presence is dependent on pattern recognition receptors (PRRs) (225). For example, toll-like receptors (TLRs) 2 and 4 work together to activate macrophages in the presence of pneumococci (225).
    At birth, macrophage levels are low with impaired phagocytosis, cell signaling, and TLR4 (discussed in Section “Innate Immune Responses” iii) expression (237, 245). Within days post birth, macrophage levels and function improve to reach adult levels (15). By contrast, with old age alveolar macrophage concentrations are depleted, cytokine production and phagocytotic activity are reduced, and lowered expression of MARCO contributes to poor killing of *S. pneumoniae* (15, 28, 237).
- *PRRs*: these receptors can be found on host cell surfaces that recognize PAMPs (16, 219), PRRs can also be located intracellularly or be secreted (16). PAMPs are structures found in bacteria and viruses. Many of these are necessary for virulence in pathogens. There are two main types of receptors that participate in the host’s immune response to pneumococcus: TLRs and nucleotide-binding oligomerization domain (NOD)-like receptors (NLRs) as described below.
  - *TLRs*: TLRs are mostly found on cell surfaces as membrane-bound molecules that recognize PAMPs (246). Recognition of PAMPs activates TLR signaling pathways that cause the recruitment of immune cells and cytokines production (247). There are currently 10 identified TLRs in humans (248). The main TLRs involved in pneumococcal infections are TLR2, TLR4, and TLR9. TLR2 is necessary in pneumococcal infection because it recognizes bacterial cell wall constituents. Former findings suggested that TLR2 recognized LTAs (16, 249, 250). However, TLR2 is now found to be binding to pneumococcal lipoproteins and peptidoglycan (225, 251, 252). TLR2 also has a role in transmission of pneumococci. Mouse models with deficient TLR2 had increased inflammation and shedding (253). TLR2 forms dimers with TLR1 and TLR6 which assist in the recognition of microbial antigens (246). TLR4 was the first TLR to be characterized and is needed for recognition of pneumococcal pneumolysin (51, 249, 254). On the other hand, TLR9 is intracellular and senses bacterial DNA within endosomes. TLR9 binds to CpG motifs (246) on the DNA, and when activated it also has signaling pathways which result in the release of cytokines (255, 256). TLR1, 2, 4, 6, and 9 work in a myeloid differentiation primary response 88 (MYD88)-dependent manner. MYD88 is an intracellular protein necessary for signal transduction and activation of TLR signaling pathways (246, 257). In addition to cytokine production, the activation of these TLRs facilitates the secretion of co-stimulatory molecules (246) which are necessary for activating T cells (258). Thus, the functions of these TLRs also play a role in adaptive immunity (Figure 9) (246, 255, 256).
    Aging greatly affects TLR function. Expression of TLR1 is reduced with age (28). TLR4 expression appears to remain normal but experiences a reduction of function (15, 28). This association has been made in mice, due to macrophages having a lowered production of pro-interleukin-1B (15, 28, 259, 260). This also indicates TLR4’s inability to respond to pneumoccocal cell wall components (261, 262). Overall TLR cell signaling impairment causes a reduction in cytokines produced, leading to poor defense against *S. pneumoniae* (28).
  - *NLRs*: NLRs are intracellular proteins that can stimulate nuclear factor-kappa B (NF-κB) (263), control inflammation, and activate inflammasome formation (56, 264). NOD2’s role in pneumococcal infections has been thoroughly investigated (263–265). This NLR recognizes muramyl-dipeptide which is a fragment of bacterial peptidoglycan in the cytosol (16, 22). It promotes the production of cytokines and activation of nucleotide-binding domain and leucine-rich-repeat-containing protein 3 (*NLRP3*) genes (56). For example, when NOD2 senses peptidoglycan, CCL2 is made and that recruits macrophages and monocytes to the infection (266). This is dependent on the lysozyme producing these peptidoglycan fragments (266). NLRs expression decreases with age and responses to *S. pneumoniae’s* PAMPs are weakened (261). Lack of NLR expression may contribute to the chronic low pro-inflammatory state observed in the elderly (discussed in Section “Additional Immune Response Considerations”).
  - *CD14*: this has been characterized as a PRR as it recognizes LTA and other cell wall components (247, 267). CD14 works by interacting with other PRRs such as TLR4 for signal transduction (267). It has been reported that, in the case of pneumococcal infections, CD14 promotes growth and dissemination of the bacteria (22, 267). Previous studies have found CD14 to be beneficial and protective to hosts against Gram-negative infections, but as for Gram-positive pathogens such as pneumococci, it instead enhances the pathogenesis of the bacteria and facilitates infection (267).

### Adaptive Immune Responses (B and T Cells)

Adaptive immune responses transpire a few to several days post-infection. The cells involved in adaptive immune responses respond to specific antigens from pathogens. Adaptive immunity can also be broken down into two types of responses: humoral and cell-mediated (268). Humoral immunity involves B cells that are activated by antigens, and production of antibodies that are specific to antigens. Cell-mediated immunity also involves T cells, including T cell activation and T cell-mediated recruitment, which involves the activation of other immune cells that can directly kill pathogenic cells (268).

These immune cells are formed in the bone marrow—B cells mature in the bone marrow into plasma cells that make antigen-specific antibodies (268). Infections at mucosal sites are controlled by the pneumococcal specific IgA antibody. IgA is observed in mucosal areas of the nose and saliva following *S. pneumoniae* colonization (269). Secretory IgA is important for opsonizing *S. pneumoniae* and promoting phagocytosis (230). *S. pneumoniae* on the other hand, possess an IgA1 protease the cleaves the IgA (discussed in Section “Virulence Factors”). This blocks opsonization. Following cleaving, the remaining Fab fragment binds to the cell wall (230, 270). This exposes CBPs, decreases negative charge of the capsule and increases cell adhesion (230, 270). Studies suggest that the Fab neutralizes the negative charge of the capsule and instead promotes cell adhesion (230, 270). Furthermore, the complement (C3) activates B cells. Following antigen stimulation, the naïve B cells differentiate into IgM^+^ memory B cells. Class switching produces other immunoglobulins needed for clearing the infection (269).

T cells instead migrate to the thymus for maturity into mature helper (CD4^+^) and cytotoxic (CD8^+^) T cells (268). Antigen-presenting cells (APCs) paired with the major histocompatibility complex (MHC) proteins present antigens (specifically, peptides) to T cells to stimulate an immune response (268). In pneumococcal infection, CD4^+^ T cells are stimulated *via* co-stimulatory molecules and APCs. Upon activation, helper T cells differentiate into Th1 and Th2 cells (Figure 9). Th1 helper cells stimulate a cellular-mediated immune response by producing cytokines such as interferon-gamma (IFN-γ), that activate and recruit other immune cells such as macrophages (271). Th2 helper cells release IL-4 cytokines, and are geared toward facilitating a humoral immune response (271) by interacting with B cells and aiding in antibody production (268). Cytotoxic T cells directly kill infected cells (268). Furthermore, upon activation of T and B cells, they can differentiate into memory B and T cells that can provide quicker clearance in reoccurring infection (268). Similarly, natural killer T-cells are also important for clearance of pneumococci (16, 22). More specifically, CD4^+^ T cells have been found to provide protection to *S. pneumoniae* in an antibody-dependent manner (272). T-helper 17 (Th17) and regulatory T cells (Tregs) are also very important for pneumococcal infections. Th17 cells release the cytokine IL-17 which is pro-inflammatory. IL-17 is needed for recruiting and activating macrophages, monocytes, and neutrophils to sites of infection and promotes clearance of *S. pneumoniae* (273). Increased production of IL-17 has been connected to reduced *S. pneumoniae* density in the nasopharynx of mice and children (273). Tregs are necessary for regulating Th17’s production of IL-17. Imbalance between Tregs and Th17 cells can lead to autoimmune disease due to over inflammation (273).

Infants experience poor T cell responses to foreign antigens because their exposure to non-maternal antigens was restricted prior to birth (237). Infants also display a skewed Th2 response to foreign antigens. To compensate for this, infants have a population of γδ T cells that generate IFN-γ, to provide a Th1 type response (237). As for B cells, in infants there is a limited response to antigens due to low expression of co-receptors (237). Infants also experience incomplete class switching for immunoglobulins and lower somatic hypermutations compared to adults (237). Immunoglobulin protection against *S. pneumoniae’s* capsular polysaccharides is developmentally regulated. At birth, maternal IgG antibodies protect infants until 27 days of age (based on the half-life of IgG) (28, 274). Once maternal antibodies have been depleted, the infant’s ability to protect itself *via* steady antibody generation experiences a delay until age two (28). By contrast, IgM has been detected in infants following *S. pneumoniae* infection and carriage (28, 275, 276). Encountering the pathogen again, also promotes antibody production similar to booster effects in vaccines (discussed below) (277). With development, the adaptive immune cells mature, develop memory, and the incidence of *S. pneumoniae* infections decrease.

In elderly populations, the efficacy of the adaptive immune cells diminishes. Aging leads to reduced production of antibodies, immunoglobulin class switching, and cell maturation, which promotes *S. pneumoniae’s* colonization (275). Antibodies specific for capsular polysaccharides decrease with age (275). In addition, there is an overall reduction in naïve T cells with age due to thymus involution (278). Previously Th17 levels were described to increase in elderly populations whereas most recently, in 2014, van der Geest et al. showed lower Th17 concentrations and increased concentrations of memory Tregs (15, 279). The ratio between CD4^+^ and Treg cell populations was also reported to increase toward more Tregs (15, 279). Diminished responses from the adaptive immune cells explain the higher incidence rates of pneumococcal diseases in these high-risk age groups.

### Additional Immune Response Considerations

#### Chemokines and Cytokines

These are signaling molecules released by innate and adaptive immune cells and receptors to direct other immune cells to the infected tissues (242, 268). Chemokines are examples of cytokines that attract cells to the infected site. In addition to recruiting cells, they promote inflammation (242, 268). TNF-α, a well-studied pro-inflammatory cytokine, inhibits growth and dissemination of pneumococci (22). Together TNF-α and IFN-γ can enhance clearance of pathogens by activating phagocytes. T cells, monocytes, and macrophages produce TNF-α (158). The phagocyte-activating cytokines are suggested to be inhibited by autolysin activity in pneumococci (158).

The elderly experience chronic low-grade age-associated inflammation (inflammaging) (28). This involves constant low levels of pro-inflammatory cytokines such as TNF-α and IL-6. The inflammatory state of the elderly is worsened due to increased NF-κB activation and the secretion of pro-inflammatory cytokines such as TNF-α from senescent cells (28). High concentrations of TNF-α have been correlated with higher disease incidences (28). Inflammaging induces the expression of host proteins which enhances *S. pneumoniae* adhesion, and is often accompanied by other morbidities that increase risk of *S. pneumoniae* infections (28).

#### Inflammasome

This is a protein complex that consists of a sensor protein, caspase 1, and an apoptosis-associated Speck-like protein with a caspase recruitment domain (ASC) (280). The inflammasome is used by the host for indirectly recognizing bacterial or pathogenic molecules and DNA (56). Upon recognition, the inflammasome regulates cytokine production (56). NLRP3 plays a role in identifying pneumococcal infection, activating macrophages and has been shown to directly interact with pneumolysin during pneumococcal infection (225). Pneumolysin can directly activate NLRP3 (281), and when activated, inflammasomes secrete IL-1β and IL-18 (282). Although inflammasomes may aid in the recognition of pathogens, the activation of inflammasomes promote inflammation and this can be harmful to the host (56).

Cho et al. studied the effects of aging on NLRP3’s activation in mice (261), and reported that enhancement in ER stress with age leads to decreased NLRP3 inflammasome activation in *S. pneumoniae* infection. Ensuring that NLRP3 is activated appropriately in the elderly population will promote stronger immune defenses against *S. pneumoniae*.

#### Complement System

This is comprised of a set of small proteins that enhance the ability of antibodies and phagocytic cells to clear microbes and damaged cells (219). These proteins can mark antigens and cells by coating them with opsonins (16, 268). Complement activation involves three cascade pathways: classical, mannose-lectin, and alternative pathways. In the classical pathway, the complement proteins bind to an antibody-antigen complex (159), whereas the alternate and mannose-lectin pathways, bind directly to PAMPs and cell surface components. Which pathway plays the main role in response to pneumococcus has been debated. Brown et al. in 2002 stated that the classical pathway is most important in response to pneumococcal infection following investigations of complement pathways deficient mice (283). On the other hand, in 2012 Ali et al. stated that the mannose-lectin pathway is more important, after following the use of mannose-lectin pathway in deficient mice that could still use the classical and alternate pathways showed susceptibility to pneumococcal infection (284). The importance of the different complement pathways’ role in pneumococcal infections needs to be further investigated. However, irrespective of the specific pathway, the complement proteins also help to fight infections by pathogens such as pneumococcus by promoting inflammation, attacking pathogens, and rupturing their cell walls (16, 22, 219). For example, mice deficient in complement C3 infected with *S. pneumoniae* were unable to clear the infection and had short survival times in comparison to mice with complement C3 (285). *S. pneumoniae* can evade the host complement system in many ways—most of which were previously discussed in the Section “Virulence Factors.” Pneumolysin is able to divert the complement system away from *S. pneumoniae* by directly activating the classical complement pathway (285). PspA inhibits C1q binding and polyhistidine triad proteins are suggested to degrade C3. *S. pneumoniae’s* complement evasion has been detailed by Dockrell and Brown (225).

The effects of aging on the complement system are complex. Previous studies suggest that complement levels are low in infants (286, 287). In 2014, Grumach et al. also showed that in newborns complement activity is low with C1, factor H, and C3a levels being lower than adult levels (287). Studies have also indicated that complement activity is greater in the elderly compared to young adults (15, 241, 288).

#### Acute Phase Serum Proteins

These proteins increase in concentration within the blood during an acute inflammatory infection (289). The three main proteins that have been investigated and associated with pneumococcal infection include C-reactive protein (CRP), serum amyloid P (SAP), and mannose-binding lectin (MBL) (289). These proteins work to alleviate infections and can recognize and bind to bacterial surfaces (289). Acute phase proteins are made as a result of cytokine production from innate cells such as macrophages (289). For example, CRP production by the liver is increased in response to IL-6 (289). CRP and SAP bind to PCho which is part of the *S. pneumoniae’s* cell wall. Once bound to the PCho, CRP and SAP activate the complement deposition on the bacteria *via* the classical pathway (290). As for MBL, there are conflicting reports about its role in pneumococcus infection as discussed above in the description of the complement system. It has been shown to recognize and attach to sugars on the cell surface of *S. pneumoniae* (291), but more verification is needed on MBL’s role in pneumococcal infection.

## Diagnosis, Age-Dependent Response, Prevention, and Disease Prognosis

### Diagnosis

Currently, there are several methods utilized in pneumococcal disease diagnostics. Traditionally, diagnosis begins with physicians performing a physical exam. For example, in the case of an ear infection, an otoscope (24) is used to confirm infection, whereas for pneumonia physicians monitor breathing for cracking sounds and wheezing (1, 292, 293). More specifically, for pneumonia, based on the results of the physical exam, physicians can conduct a chest X-ray to examine the lungs and monitor inflammation to confirm the presence of infection (292, 293). This X-ray is also performed following signs of respiratory distress (293, 294). Blood oxygen levels are also measured *via* pulse oximetry in both children and adults to assess the severity of the infection (1, 292, 293). Pulse oximetry at the primary care level should be the future, and future technological developments might add respiratory rate and work of breathing to the parameters measured by oximetry (295). Body fluids are also processed to assess whether or not pneumococcus is present, and to confirm its identity (1, 292–294, 296). These fluids include blood, urine, cerebrospinal fluid, and saliva (1, 292–294, 296). The blood test allows physicians to examine complete blood cell count. This test confirms whether or not an infection is present by giving an estimate of the percentage of WBC that are circulating (1, 292–294, 296). A large concentration of WBC is indicative of an infection (1, 292–294, 296). This is expected in infection response. However, Gardner et al. in 2017 indicated that upon the time of admission, about 25% of subjects with pneumococcal pneumonia and roughly 38% with CAP actually have normal WBC counts (297). Studies have also shown that poor prognosis has been associated with low WBC (297). New findings associate low WBC rather than high WBC with poor prognosis (297). These conflicting results indicate that WBC count alone should not be used to diagnose pneumonia and should be better investigated as key indicator of pneumonia.

Bacterial cultures and Gram-staining tests using body fluids are important for determining the strain of bacteria and confirming its identity (1, 292–294, 296). Currently, physicians are investigating other means of diagnosing pneumococcal infections due to the poor yield and quality of sample when conducting cultures. This process is also dependent on bacterial growth which can be time consuming. One useful tool that is being developed is the urinary antigen detection test (298), which is only currently used in adults. This test monitors the levels of the C-polysaccharide antigen of pneumococcus in the urine. It appears to be quicker, can allow for targeted treatment with better results than culture-based methods of diagnosis (1, 292, 293, 298). In addition to testing for pneumococcus, physicians also test for other bacteria which may be causing the infection, and other viruses such as influenza which can coinfect patients (293). Once all the tests confirm the presence of an infection, the cause of the infection and the severity of the disease patients are treated accordingly.

Currently, thoracic ultrasounds are being investigated as a method for diagnosing CAP (299). When compared to chest X-rays, thoracic ultrasounds identified 73.5% of the lung consolidations confirmed by chest X-rays, with about 27% false negative results. D’Amato et al. suggest using ultrasound as a monitoring tool. Lung ultrasound has been tested for its diagnostic potential and it was found to be a sensitive tool for confirming CAP in children (300). 96% children with pneumonia were detected, however, given the small sample size, further investigation is necessary. Chest computed tomography is not used for children due to radiation (300). Recently, a computer-aided differential diagnosis system was tested for distinguishing types of pneumonia, using high-resolution computed tomography. This method was compared to radiologists’ classification of interstitial and non-specific interstitial pneumonia, and was concluded to be a robust method for diagnostics (301). In addition, researchers have proposed combining clinical signs and laboratory markers to assess an individual’s risk of contracting pneumonia. For example, high levels of CRP and procalcitonin accompanied by unilateral hyperventilation and grunting were associated with pneumonia (302). On the other hand, children with no clinical signs of pneumonia and low CRP results were at a lower risk for pneumonia. The use of PCR for diagnosis is also being developed. A positive blood pneumococcal PCR can more accurately confirm the diagnosis of pneumonia (302). PCR has been used to detect pneumolysin in whole blood samples (303). The sensitivity of PCR tests varied from 68 to 100% and had poor specificity (303). By contrast, assessment of quantitative real-time PCR indicated that it is more successful in achieving greater speed, specificity, and sensitivity compared to multiplex PCR (304).

### Prevention, Antibiotic Response, and Age-Dependent Immune Responses

The two main modes of preventing pneumococcal infections are using antibiotics and vaccinations against pneumococcus (24). Antibiotics are essential in reducing bacterial load (305). Such treatment can work by killing the bacteria or hindering their growth (305). The first antibiotic to be created was penicillin which was discovered in 1928 by Alexander Fleming (306), and antibiotics have been used widely since. However, misuse of antibiotics can cause bacteria to become resistant (39, 305, 307). Resistant bacteria are then able to survive post antibiotic treatment and they can grow, multiply, and share antibiotic-resistant genes with each other. Pneumococcal strains that were penicillin-resistant were first recorded in the 1970s (22). Currently, penicillin-resistant strains have spread worldwide with pneumococcus also being resistant to other types of antibiotics: erythromycin, tetracycline, and chloramphenicol (305). *S. pneumoniae* acquires multiple antibiotic resistance genes *via* transformation and evolution with the increase in antibiotic use (308). Mutations in penicillin-binding proteins (*pbp*) affect binding of penicillin which acts by blocking cell wall synthesis (309). Erythromycin resistance gene *erm(B)* blocks the binding of macrolides (antibiotics targeting protein synthesis) and *mefA* and *mefE* genes produce an efflux pump which regulates entry of the antibiotics (308–312). Resistant *S. pneumoniae* strains have rapidly spread, and infections are harder to treat. In 2013, the CDC estimated that about 30% of pneumococcal cases were due to antibiotic resistance to one or more antibiotics (307). This resistance increases the number of doctor visits and hospitalizations (307). For example, the CDC reports that the resistance can lead to 1,200,000 more illness and 7,000 deaths annually (307). This reduction in ability to treat and clear the pathogen led to the development of vaccines that would provide protection prior to infection and thus reduce the need for antibiotics (305).

Currently, there are two types of inactivated vaccinations that protect against *S. pneumoniae* (313–315). The pneumococcal polysaccharide vaccine 23 (PPSV23) (316) uses purified capsular polysaccharides and is routinely given to adults who are 65 and older (313–315). It protects against 23 serotypes of *S. pneumoniae* and is effective in 50–70% of cases in adults (126). This vaccine works in a T-cell independent manner. The polysaccharide antigens are recognized by B cells which differentiate into plasma cells that produce antibodies specific for the polysaccharide antigens (317). PPSV23 provides T-cell-independent immunity and requires revaccination 5 years after the first vaccination because the immunity is transient (316, 318). The pneumococcal conjugate vaccine (PCV) (319) was developed after noticing the low efficacy and poor immunogenicity of PPSV23 in infants and young children (91, 320). In the conjugate vaccine, the purified polysaccharides covalently conjugated to a carrier protein, specifically CRM197 (318, 319). The current FDA approved conjugate vaccine is PCV13 which protects against 13 serotypes of the *S. pneumoniae* (319). PCV13 replaced PCV7 in 2010 and protects against six additional serotypes (321). This elicits a T-cell-dependent response which provides mucosal immunity and immunologic memory in children (126). PCV13 provides long-lasting immunity by causing B and T cells to interact (317). B cells recognize and process the carrier protein (317). The MHCII needed for antigen-presentation to T cells, binds to the peptide produced following B cell breakdown of the carrier protein (317). The peptide is presented to the T cells by MHCII at the surface of the APC, providing co-stimulation necessary for producing plasma cells and memory B cells (317). The use of this vaccine has led to a decrease in pneumonia cases in young children by more than 90%, and is most effective in children younger than five (126).

When it comes to high-risk individuals, the CDC recommends the prime-boost method of vaccination. This involves priming the immune system to a specific antigen, and enhancing this antigen-specific immune response by re-administering the antigen (322). The prime-boost strategy increases immunity to antigens and is recommended for high-risk individuals (323, 324). There are two ways to prime and boost the immune system: homologous, in which the same vaccine is received twice, and heterologous, which utilizes different types of vaccines (322). The heterologous method has been shown to be more immunogenic (325). Currently, children and adults who are at high risk for pneumococcal disease and have pre-existing conditions undergo the prime-boost strategy prevention by receiving the PCV13 followed by the PPSV23 (326, 327). This is also due to the poor immunological response seen in HIV patients who receive the PPSV23. Prime-boost vaccinated HIV-infected groups have been shown to be more likely to display a twofold increase in IgG geometric mean concentrations (328). PCV13 provides a longer and stronger level of protection against *S. pneumoniae* (323). Within 4–8 weeks, PCV13’s IgG levels can equal or exceed PPSV23 in high-risk individuals (323, 324).

Despite the availability of pneumococcal vaccines, it is important to note that these vaccinations are both serotype and age dependent (23, 91, 313–315, 320). Understanding the role that age plays in host immune system activation is essential for better prognosis and treatment of diseases. As stated previously, young children and the elderly are at higher risk for contracting pneumococcal diseases (237). This is due to immunosenescence within the elderly population, whereas for infants, it is due to their underdeveloped immune systems (237). In addition to age recommendations, the CDC also recommends the use of either vaccine in high-risk individuals with pre-existing health conditions. For example, both vaccines are recommended in young children and adults ages 19–64 with pre-existing health conditions (23, 24, 91). PPSV23 is also recommended by the CDC for use in adults that smoke or have asthma (24). These vaccine recommendations are reevaluated regularly based on vaccine efficacy and changes within the bacteria serotypes (326, 329).

Vaccines have drastically reduced invasive pneumococcal diseases, especially CAP in young children and adults (Table 2) (18). However, these vaccines have pitfalls. First, there have been at least 97 serotypes identified but these vaccines protect against 14–25% of these. The current vaccines only protect against *S. pneumoniae* serotypes that are mainly associated with causing the disease. Some studies suggest that there is little evidence that PPSV23 protects against non-invasive pneumococcal diseases, which are more prevalent in adults (330). The CDC also confirms that PPSV23’s efficacy in non-bacteremic pneumonia has led to contradicting findings, but nevertheless, it has shown sufficient efficacy in invasive pneumococcal diseases (331). Weinberger et al. discuss the challenges of vaccinating the elderly with PCV13 and PPSV23. These researchers argue that PPSV23 does not show a real benefit to the elderly (330). As for, PCV13 they argue that it is already used in children and thus adults should be partially protected from serotypes in PCV13 due to herd immunity (330). They also state that herd immunity should provide partial protection and thus will lead to reduction of efficacy of PCV13 (330). Other studies also discuss herd immunity from PCV13 due to infants and toddlers being vaccinated (332, 333). Due to PCV13, disease serotypes rates within this vaccine will decrease by 50% (332, 333). This becomes a problem because of serotype replacement. The serotypes that are not in the vaccine can colonize young children and spread to adults (334, 335). In addition, with serotype vaccines, the serotypes that are popular and commonly cause CAP and other diseases may not necessarily do so in the future and so these vaccines would need to be reevaluated. PCV13’s replacement of PCV7 was a prime example of changes to serotypes that cause pneumococcal diseases. Recently researchers at Merck Sharp and Dohme Corp. completed a phase 1 clinical trial (NCT01215175) investigating a new conjugate vaccine, PCV15, immunogenic, and safety properties compared to PCV13 (336). This contains two extra serotypes (22F and 33F), which were previously identified for the cause of approximately 10% of invasive pneumococcal diseases in adults in 2007 (337). Another concern for current vaccines is that 3–19% of pneumococcal diseases are due to non-encapsulated *S. pneumoniae* (38). Current vaccines are ineffective against non-encapsulated *S. pneumoniae* due to serotype specificity (38). Further developments of vaccinations are vital for eliminating the burden of *S. pneumoniae* and reduce the number of infections.

### Post-Infection Prognosis

Following pneumococcal diseases such as pneumonia, high-risk individuals may experience longer recovery times and complications due to the disease (1, 292, 293). About 1.6 million deaths from pneumococcal diseases occur worldwide (36). According to the CDC, there were over 50,000 deaths within the US during 2014 (29) and the majority of these deaths were seen in the elderly (29). Older adults have lower survival rates than other age groups (94, 338). The elderly may recover from pneumococcal diseases such as CAP, but they face higher death rates due to the high possibility of developing other health problems and the reoccurrence of the disease (1, 94, 338). Infants and young children who recover from CAP have an increased risk for developing respiratory problems (339). For example, research indicates that young children face a greater risk for reduced lung function and developing chronic obstructive pulmonary disease (44, 339). In some cases, increased death rates and complications are due to delays in diagnosis. Such delays in turn hamper timely treatment, which also increases the severity of the disease. For example, meningitis can progress quickly and cause permanent disabilities such as brain damage, hearing loss, and seizures (24, 52, 53). Timely treatment can reduce the risk of neurological damage and death due to this infection (53). In addition, ear and sinus infections can lead to hearing loss and respiratory problems respectively (24). The environment also plays a role in affecting recovery rates and reoccurrence of the disease especially for smokers and those residing in nursing homes and crowded areas (1). Furthermore, Tiewsoh et al. study investigating the outcome of children with severe pneumonia showed that children who were not breastfed had a low birth weight and were within crowded homes had longer hospital stays and the initial antibiotics were not helpful and required new antibiotics (340). Nutrition also plays a vital role in how well someone will recover from these diseases (341).

Some complications due to pneumococcal pneumonia include respiratory failure, lowered oxygen levels, and collapsed lungs (4). It is also possible for the lungs to fill with fluid and this fluid can become infected. *S. pneumoniae* may also migrate to the blood (4, 24). This is called bacteremia which is the most common complication for pneumonia (4, 24). Pneumonia and other pneumococcal diseases are classified as invasive if the bacteria migrate to the blood. In addition, individuals with this disease can develop pericarditis which is inflammation of the sac around the heart, lung abscess, empyema, and blockage of airways (4, 24). It is also highly probable for coinfections to occur when suffering with pneumonia. An example of this is influenza—66% of CAP cases also present coinfection with influenza (113). Most of these health complications are seen in elderly subjects, and this also points to the increasing importance of improved diagnostics, treatments, and vaccinations for this age group.

## Discussion

Pneumococcal diseases cause millions of deaths worldwide (36, 97). In this review, we have characterized *S. pneumoniae*, explored its virulence factors, and how hosts respond to its presence. We have discussed the host defenses against *S. pneumoniae*, and how individuals with weakened immune systems may experience a harder time clearing the pathogen. We have also indicated that young children, elders, and individuals who are immunocompromised all have an increased risk for contracting pneumococcal diseases. The majority of previous efforts have provided an extensive characterization of *S. pneumoniae* features and began probing the interactions of the bacteria with the host in the context of pneumococcal disorders. However, in terms of treatment and prevention there remain substantial open questions that need to be addressed as discussed below.

There are a variety of methods available for pneumococcal disease diagnostics. Many of the current tests needed to confirm *S. pneumoniae’s* identity are culture-dependent (1, 292, 293). Culture-independent methods that take advantage of the latest technologies are being developed, such as the use of a lung ultrasound to assess pneumonia (342). Chavez et al. and Long et al. discuss the possibility of lung ultrasound use in pneumonia diagnosis (343, 344) indicating high diagnostic accuracy, while at the same time providing a radiation free method of examining the lungs (343, 344). Similarly, the use of mass spectrometry to examine metabolites from the saliva (345), breath (346), and urine (298, 347) of patients being tested for pneumococcal diseases is under development. The urine antigen test discussed above also provides rapid results that will allow for quicker diagnosis and treatment once *S. pneumoniae* antigens are detected in the urine (298, 347).

With diagnostic methods improving, pneumococcal disease treatments are also being updated. Antibiotics are available to reduce the colonization of *S. pneumoniae*, however, the efficacy of antibiotics is being reduced due to the increase in antibiotic resistance (305, 307). Broad-spectrum antibiotics are no longer as effective (305, 307). Inhaled therapeutics are underdeveloped but can be beneficial for treating pneumonia and other pneumococcal diseases. This method can provide a mode of delivering antibiotics and antimicrobials (348) in a more targeted manner, improve mucociliary clearance *via* hypertonic saline solutions (348) and inhalation of cytokines to stimulate the immune system (348). On the other hand, to also reduce the effect of antibiotic resistance, *S. pneumoniae* strains may also be studied *via* RNA-sequencing and other high throughput technologies to detect antibiotic resistance genes and thoroughly characterize serotypes.

Treatment and prevention of pneumonia and other pneumococcal diseases are of major concern for the clinical field due to the high death rates and low efficacy of current vaccines due to aging differences and serotype replacement. Some alternative vaccination methods have been proposed and are also being developed. For instance, Weinberger et al. propose the use of a conjugate vaccine that is specific for elderly subjects, which targets the serotypes not in current vaccines but other serotypes that are mostly seen in elderly patients with pneumococcal diseases (330). Some researchers have proposed creating a conjugate vaccine that targets all or more of the identified serotypes of *S. pneumoniae* (330, 349). However, the impact on the immune system and immunogenicity of this vaccine would need to be thoroughly investigated (330). This vaccine would also need to demonstrate better efficacy than existing vaccines (330). In addition to this, conjugate vaccines are expensive [currently, the PCV13 costs about $160 per dose (350)], and a true benefit will need to be clearly identified. In addition, observing how pneumococcal disease incidence rates are changing as more and more people are getting vaccinated will lead to accurate assessment of pneumococcal disease burden and vaccine efficacy (341). Vaccination policies and cost-effect analyses can benefit from information on vaccine disease reduction (341).

Serotype-independent vaccines are also being investigated. These include protein, protein and polysaccharide combination, and whole cell vaccines (330, 349, 351, 352). Protein vaccines would contain surface proteins that are highly conserved in *S. pneumoniae* (353, 354). For example, PspA and inactivated pneumolysin have been tested in phase 1 clinical trials as protein antigens (354). They both demonstrated safety (354), but PspA antigen’s immunogenicity was low (355) whereas the inactivated pneumolysin was found to be immunogenic and effective in eliciting protective immune response (356). PspA is considered an ideal protein candidate because reports indicate that PspA family 2 is commonly found in *S. pneumoniae* strains (357). For example, in Pakistan most strains of pneumococci have PspA genes (357). These protein vaccines can provide an extra preventative method once developed and will require thorough analysis of regulation and what regulatory issues may be faced (358). In addition, as a form of combination therapy, a vaccine with protein antigens as well as conjugated polysaccharide antigens may also provide a broader range of protection against pneumococcal diseases (353, 354). On the other hand, whole cell vaccinations would introduce a dead *S. pneumoniae* cell to hosts with the potential to provide broader protection to *S. pneumoniae* (359, 360). HogenEsch et al. investigated the use of whole cell vaccines in mice by using a capsule deficient and autolysin mutant cell (359). This exposed the host to multiple parts of *S. pneumoniae*. They found that the vaccine led to the productions of antibodies and IL-17 which defend against *S. pneumoniae* colonization of the nasopharynx in mice (359). Researchers have also started developing live attenuated pneumococcal vaccines (361, 362). The SPY1 strain is a live attenuated strain of pneumococci that does not have a capsule (362). Xu et al. in 2015 experimented with delivering this vaccine intranasally in mice and observed that it elicited a humoral response (362). More recently, Zhang et al. added a mineralized shell to SPY1 to improve its stability and test if it can elicit a stronger immune response (361). The modified strain (SPY1Δ*lytA*) also did not have autolysin activity (361). This modified SPY1 vaccine led to higher stability, more production of IgG, and an overall increase in protection when compared to the SPY1 vaccine (361). Additional concerns of serotype-independent vaccines include determining if the vaccines will be immunogenic in all ages, whether or not the vaccines would elicit a strong immune response, and ensuring that they can induce a pro-inflammatory state while not leading to an over activation of the immune system. All of these novel methods show great promise, but they require further assessments.

Overall, there has been progress in our understanding of pneumococcal diseases over the last three decades, however, the diseases still constitute a big burden on health care. There has been a great decrease in pneumococcal diseases since the implementation of purified polysaccharide and polysaccharide conjugate vaccines, but over time due to serotype replacement, antibiotic resistance, and changes in immunity with age, the treatments, and vaccines in place may prove ineffective. Therefore, ongoing research to improve vaccinations and treatments must continue toward alleviating the ill effects of *S. pneumoniae*.

## Author Contributions

All authors listed have made a substantial, direct, and intellectual contribution to the work and approved it for publication.

## Conflict of Interest Statement

The authors declare that the research was conducted in the absence of any commercial or financial relationships that could be construed as a potential conflict of interest.
